# The integrated landscape of causal genes and pathways in schizophrenia

**DOI:** 10.1038/s41398-018-0114-x

**Published:** 2018-03-15

**Authors:** Changguo Ma, Chunjie Gu, Yongxia Huo, Xiaoyan Li, Xiong-Jian Luo

**Affiliations:** 1 Key Laboratory of Animal Models and Human Disease Mechanisms of the Chinese Academy of Sciences and Yunnan Province, Kunming Institute of Zoology, Chinese Academy of Sciences, Kunming, Yunnan 650223 China; 2 Center for Excellence in Animal Evolution and Genetics, Chinese Academy of Sciences, Kunming, Yunnan 650223 China

## Abstract

Genome-wide association studies (GWAS) have identified more than 100 loci that show robust association with schizophrenia risk. However, due to the complexity of linkage disequilibrium and gene regulatory, it is challenging to pinpoint the causal genes at the risk loci and translate the genetic findings from GWAS into disease mechanism and clinical treatment. Here we systematically predicted the plausible candidate causal genes for schizophrenia at genome-wide level. We utilized different approaches and strategies to predict causal genes for schizophrenia, including *Sherlock*, *SMR, DAPPLE, Prix Fixe*, *NetWAS*, and *DEPICT*. By integrating the results from different prediction approaches, we identified six top candidates that represent promising causal genes for schizophrenia, including *CNTN4, GATAD2A, GPM6A, MMP16, PSMA4*, and *TCF4*. Besides, we also identified 35 additional high-confidence causal genes for schizophrenia. The identified causal genes showed distinct spatio-temporal expression patterns in developing and adult human brain. Cell-type-specific expression analysis indicated that the expression level of the predicted causal genes was significantly higher in neurons compared with oligodendrocytes and microglia (*P* < 0.05). We found that synaptic transmission-related genes were significantly enriched among the identified causal genes (*P* < 0.05), providing further support for the dysregulation of synaptic transmission in schizophrenia. Finally, we showed that the top six causal genes are dysregulated in schizophrenia cases compared with controls and knockdown of these genes impaired the proliferation of neuronal cells. Our study depicts the landscape of plausible schizophrenia causal genes for the first time. Further genetic and functional validation of these genes will provide mechanistic insights into schizophrenia pathogenesis and may facilitate to provide potential targets for future therapeutics and diagnostics.

## Introduction

Schizophrenia is a severe mental disorder with complex genetic architectures^1^. Recent studies have showed that different types of genetic variants (including common variants such as single nucleotide polymorphism, copy number variants, rare structural variants, de novo mutations and rare disruptive variants) are involved in the etiology of schizophrenia^2–10^. Though schizophrenia has a complicated genetic underpinnings, high heritability strongly suggests the pivotal role of inherited variants in genetic predisposition to schizophrenia^11^. To identify the inherited risk variants for schizophrenia, multiple genome-wide association studies (GWAS) have been performed in different continental populations and numerous risk variants (loci) have been uncovered^2,12–16^. In 2014, the schizophrenia working group of the psychiatric genomics consortium (PGC2 release) reported the largest GWAS of schizophrenia so far^2^. Despite the great success of schizophrenia GWAS and more than 100 independent risk loci have been identified, how to translate genetic findings into molecular risk mechanisms remains a major challenge.

Several key steps are needed to elucidate the genetic and pathophysiological mechanisms of schizophrenia. The first step is to identify the genetic risk variants (or loci). The second step is to pinpoint the potential causal gene (or genes) in the identified risk loci. And the third step is to investigate how the causal genes exert their effect on disease susceptibility. During the past decade, great efforts have been made and significant progress has been achieved in identification of risk variants for schizophrenia. To accelerate the discovery of novel risk variants, the PGC conducted large-scale genetic studies^2^ and over 100 schizophrenia risk loci have been reported. With the rapid increase of sample size, new risk variants (or loci) are being uncovered at an unprecedented rate and the landscape of the inherited genetic risk variants is emerging. Despite the fact that numerous risk loci have been reported, only very limited causal genes were identified in the reported risk loci. For most of the risk loci, the causal gene (or genes) that explains the association signal still remains largely unknown. In contrast to the rapid identification of risk variants, pinpointing the potential causal gene (genes) at the reported risk loci has lagged far behind. To translate the genetic risk loci into molecular risk mechanisms and to facilitate the development of new therapeutic targets, it is of great importance to identify the causal gene (s) that contributes to risk of schizophrenia in the reported risk loci.

Despite its importance in illuminating the genetic and pathogenic mechanisms of schizophrenia, localizing the causal gene remains a major challenge in human genetics. First, the risk loci identified by GWAS usually contain many highly linked genetic variants that usually span large genomic regions (sometimes up to several megabase). For example, among the reported risk loci, the MHC region showed the most significant association with schizophrenia. However, this region encompasses numerous highly linked variants spanning multiple genes (over 100). Due to the complex linkage disequilibrium (LD) between the risk variants, it is difficult to pinpoint the plausible causal gene. Second, in many cases, due to the complexity of gene regulatory^17–21^, the gene nearest the top associated variants is not necessarily the actual causal gene. Accumulating evidence shows that the risk variants may regulate distal gene expression through long-range chromosomal interactions^19,20^. These complicated linkage disequilibrium and gene regulatory impede the identification of causal gene in the reported risk loci. In this study, we systematically predicated the plausible candidate causal genes for schizophrenia through comprehensive integrative analyses, including integration of genetic associations from schizophrenia GWAS^2^ and brain expression quantitative trait (eQTL) data^22^ and using network-based prioritization approaches (based on brain-specific networks and functionally coherent subnetworks, i.e., shared-function or co-function networks). On the basis of genetic associations from GWAS of schizophrenia, we generated the first landscape of plausible causal genes for schizophrenia. We further showed that the causal genes are highly expressed in neurons and are enriched in synaptic transmission-related pathways. Finally, we found that knockdown of the top six causal genes suppressed the proliferation of neuronal cells. This landscape of plausible causal genes provides a start point to elucidate the genetic and pathophysiological mechanisms underlying schizophrenia.

## Materials and methods

### GWAS of schizophrenia

Recently, PCG reported the largest GWAS of schizophrenia so far (PGC2 release)^2^. In the first phase, genome-wide genotypes of 35,476 schizophrenia cases and 46,839 controls were obtained and meta-analyzed. The SNPs with *P* value smaller than 10^−6^ were replicated in additional samples. Through combining the results from the first and second phase, more than 100 risk loci were identified (*P* < 5 × 10^−8^) and most of them were newly reported. Genome-wide SNPs associations from the first phase (i.e., 35,476 schizophrenia cases and 46,839 controls) of PGC were used in this study. More detailed information about PGC, including sample recruitment and diagnosis, genotyping, quality control, and statistical analysis can be found in the original paper^2^.

### Brain eQTL data

We used the brain eQTL data reported by Myers et al.^22^ in this study. Briefly, human brain tissues (cortex) of 193 normal human subjects were obtained. All of the individuals were of European of ancestry and had no clinical history of neurologic and neuropsychiatric diseases. DNA and RNA were extracted using standard procedures. Genotyping was performed using Affymetrix GeneChip (Human Mapping 500K Array Set) and gene expression was measured using Illumina HumanRefseq-8 Expression BeadChip. PLINK was used to test the association between the genotyped genetic variants and gene expression using linear regression. More details about sample description, genotyping, expression quantification and statistical analyses can be found in the original paper of Myers et al.^22^.

### Integration of schizophrenia GWAS and brain eQTL data (Sherlock)

Most of the identified schizophrenia risk variants are located in non-coding region, suggesting these variants may exert their effects through regulating gene expression. To infer genes whose expression alteration may contribute to disease risk, He et al. developed a Bayesian statistical method (named *Sherlock*)^23^ to identify potential causal genes through combining genetic associations from GWAS and eQTL data. For a given gene, there may be several genetic variants (usually SNPs) that act synergistically to regulate the expression level of this gene (we called these expression-associated SNPs eSNPs). If a gene is not associated with disease, the eSNPs of this gene may not be associated with disease risk. However, if it is a causal gene, genetic variations at these eSNPs may alter its expression level, which may in turn influence disease susceptibility. Thus, the eSNPs of this gene may also be associated with disease as well. Significant overlap between the eQTL of a specific gene and the loci associated with the disease suggests this gene may have a role in disease pathogenesis. To predict the potential causal genes for schizophrenia, we systematically integrated genome-wide SNP associations from PGC2^2^ and brain eQTL from Myers et al.^22^ using *Sherlock* statistical framework^23^. More detailed information about *Sherlock* statistical inference can be found in the paper of He et al.^23^.

### Integration of schizophrenia GWAS and brain eQTL data (SMR)

In addition to *Sherlock*, we also used summary data-based Mendelian randomization (*SMR*)^24^ developed by Zhu et al^24^ to predict causal genes for schizophrenia. Similar to *Sherlock*, *SMR* predicts causal genes by integrating of summary data from GWAS and eQTL. However, the statistical inference of *SMR* is different from *Sherlock*. In this study, we used *SMR* to infer causal genes through integrating the genome-wide associations from PGC2^2^ and brain eQTL from Myers et al.^22^. By default, *SMR* only includes probes with at least one *cis*-eQTL that has a *P* < 5 × 10^−8^. However, due to the relative small sample size used in brain eQTL study, we also performed *SMR* analysis using lower transcript inclusion threshold (*P*_eQTL_ < 1 × 10^−5^). Genes passed *SMR* and *HEIDI* tests were inferred as plausible causal genes. More details about *SMR* method (including statistical inference, distinguishing pleiotropy from linkage and pinpointing functionally relevant genes) can be found in the original paper^24^.

### Predicting causal genes using functionally coherent subnetworks (Prix Fixe)

In contrast to *Sherlock* and *SMR*, *Prix Fixe* uses a different strategy to infer causal genes^25^. *Prix Fixe* utilizes shared-function or co-function networks to prioritize candidate causal genes. The prioritization procedure includes several steps. First, disease-associated SNPs (index or lead SNPs) were used to define linkage-disequilibrium windows. SNPs linked with the index SNP (*r*^2^ > 0.5) were identified and nearby genes were extracted. Second, *Prix Fixe* constructs a comprehensive human co-function network through extracting functional relationships between human genes. Third, *Prix Fixe* identifies the mutually connected (densely interacted) subnetworks using the co-function network. Finally, *Prix Fixe* prioritizes the candidate genes based on their importance in the subnetworks and a *Prix Fixe* score (PF score) was obtained for each candidate gene. *Prix Fixe* evaluates the importance of each gene in the defined LD windows, using a specific parameter, i.e., edge density, the number of edges in the subnetwork. If a gene has no functional interactions (edge connections) with other genes in the subnetworks, the presence or absence of this gene will not change the edge density. However, if a gene has functional relationships with other genes in the subnetwork, the presence or absence of this will affect the edge density. In this study, the top 100 index SNPs from PGC2^2^ were used as input for *Prix Fixe*. (as *Prix Fixe* only can accept a maximum of 100 SNPs as input).

### Identifying causal genes using brain-specific functional interaction network (NetWAS)

Greene et al. recently generated a genome-scale functional interaction networks for many human tissues (including brain)^26^. These tissue-specific networks can be used to prioritize potential causal genes^27^. Briefly, SNP-level association statistics were converted into gene-level statistics (i.e., gene-based *P* values), which were then integrated with tissue-specific networks to predict the potential causal genes. In this study, we first converted SNP-based summary statistics (SNP *P* values from PGC2) into gene-based *P* values using *Pascal*^28^. The gene-level *P* values were then used as input for *NetWAS*^26^, and brain-specific functional interaction networks were selected. More detailed information about tissue-specific networks and *NetWAS* can be found in the original paper^26^.

### Prioritizing causal genes using predicted gene functions (DEPICT)

To identify the genes and pathways that can explain the genome-wide associations, Per et al. developed an integrative tool (*DEPICT*) to prioritize the most likely causal genes through using predicted gene functions^29^. Briefly, *DEPICT* first predicts gene function by using co-regulation of gene expression and previous annotated gene sets. *DEPICT* then generates a “reconstituted” gene sets, which contain the likelihood of membership of each gene in the reconstituted gene sets. Finally, through using predicted gene function and the statistical significant loci identified by PGC2, *DEPICT* prioritizes causal genes for schizophrenia. More details about *DEPICT* can be found in the paper of Pers et al. As Pers et al.^30^ have predicted the potential causal for schizophrenia using *DEPICT*, we included their results (i.e., prioritized causal genes) into our study.

### Predicting causal genes using protein–protein interaction network (DAPPLE)

Previous studies have shown that proteins encoded by disease-associated genes tend to physically interacted than random expectations^31,32^, a phenomenon called “guilt by association”^33,34^. Based on the “guilt by association”, protein interactions can be used to prioritize disease-associated genes^35–38^. In this study, we used Disease Association Protein–Protein Link Evaluator (*DAPPLE*)^36^ to prioritize the plausible causal genes at the reported loci by using protein–protein interaction data.

### Ranking of the prioritized causal genes

We used different approaches to predict the causal genes. A gene may represent a promising candidate if it is predicted by different predicting methods. A cumulative scoring strategy was hence used to rank the casual genes. Briefly, each prioritization approach contributes one point to the total score of the prioritized causal genes. For example, if a gene is only prioritized by one method, the total score of this gene is one point. If a gene is identified by two methods, the total score of this gene is two points. The total score of each gene was calculated and ranked. A higher total score indicates a higher probability that the prioritized gene is causal.

### Gene ontology analysis

To investigate whether the predicted causal genes were enriched for specific functional categories, we performed Gene Ontology (GO) analysis using DAVID^39,40^. Three GO terms, including biological process (BP), cellular component (CC), and molecular function (MF) were used to test whether the prioritized genes are significantly enriched in specific biological processes or pathways. The significance (*P* values) of the overrepresented GO terms was corrected by the Benjamini–Hochberg procedure.

### Spatio-temporal expression pattern analysis of plausible causal genes

RNA sequencing-based expression data from the Brainspan^41^: Atlas of the developing human brain were used to plot the expression trajectory of the prioritized causal genes. Detailed information about sample collection, quality control, RNA extraction, and quantification can be found in the Brainspan website (http://www.brainspan.org/). We processed the data and plotted the gene expression as previously described^8^.

### Cell-type-specific expression analysis of plausible causal genes

We examined the expression of the prioritized causal genes in different cell types using data from Zhang et al.^42^. Briefly, different cell types of human brain (including fetal astrocytes, mature astrocytes, neurons, oligodendrocytes and microglia/macrophage cells) were isolated and gene expression was measured by RNA sequencing. The RNA sequencing-based expression values (FPKM, fragments per kilobase of exon per million fragments mapped) were downloaded and processed. As described previously^43^, the expression level of each gene was expressed as log_2_(FPKM + 1). In addition, we also examined the expression of the predicted causal genes in human embryonic stem cells (ESCs, line H9) and NSCs using the expression data from Lafaille et al.^44^. More detailed information can be found in the original papers^42,44^ and supplementary material. Analysis of variance (ANOVA) was used to test whether the expression level of the prioritized genes was significantly different among different cell types. Test of homogeneity of variances showed lack of homogeneity (*P* < 0.05). We thus used Dunnett C test^45,46^ (implemented in SPSS) to compare if the mean expression level of the predicted causal genes was significantly different among different cell types.

### Analysis of protein–protein interaction and co-function network

To investigate the physical interaction among the proteins encoded by the predicted causal genes, we extracted the protein–protein interaction (PPI) data from GeneMANIA (http://genemania.org/)^47^, a well-characterized PPI database that contains high-confidence interaction data. We also explored the functional relationships between the prioritized causal genes using functional-association network (FAN) from study of Tasan et al.^25^.

### Expression analysis of the top causal genes in schizophrenia cases and controls

The expression level of the top causal genes in schizophrenia cases and healthy controls was compared using expression data (GSE53987 and GSE21138) from gene expression omnibus (GEO). GSE53987 contains 15 subjects with schizophrenia and 19 healthy controls. Three brain regions (hippocampus, prefrontal cortex, and straitum) were included in GSE53987. RNA was isolated and gene expression level was quantified using Affymetrix array chips (U133_Plus2). GSE21138 includes 30 schizophrenia cases and 29 healthy controls. Brain tissues from the prefrontal cortex (Brodmann Area 46) were isolated, and gene expression was measured using Human Genome U133 Plus 2.0 array. The raw data of each study were downloaded from GEO, and Bioconductor (‘affy’ package) was used to process the data. We used RMA algorithm^48^ to normalize the expression data. Expression difference between cases and controls were tested using Student’s *t* test. More details about RNA isolation, quantification, quality control, and statistical analysis can be found in the original paper^49^.

### Knockdown of top causal genes in SH-SY5Y cell line

Human neuroblastoma SH-SY5Y cells were maintained in Dulbecco’s modified Eagle’s medium (DMEM)-F12 (1:1) containing 10% fetal bovine serum (FBS). Short-hairpin RNA (shRNA) sequences targeting to *CNTN4, GATAD2A, GPM6A, MMP16, PSMA4*, and *TCF4* were designed using BLOCK-iT™ RNAi Designer (https://rnaidesigner.thermofisher.com/). Sense and anti-sense oligonucleotides were synthesized, annealed and cloned into the PLKO.1 vector at the AgeI and EcoRI restriction sites. Knockdown efficiency was determined by quantitative PCR (qPCR). The shRNA sequences and primers used for qPCR are listed in Supplementary Material and Supplementary Table S1-S2.

### Proliferation assays

SH-SY5Y cells were plated at a density of 1 × 10^4^ cells per well in 96-well plates. After 24 h, cells were transfected with 0.2 μg shRNA constructs using lipofectamine 3000 (Invitrogen). Cell counting kit-8 (CCK8) (Sigma) was used to quantify the cell number, as previously described^50^. After 72 h transfection, 10 μL CCK8 solutions were added into each well, incubated for 6 h. Then cell amounts through measuring the absorbance at 450 nm using a micro-plate reader.

## Results

### Causal genes identified by Sherlock

We integrated the SNP associations from schizophrenia GWAS (PGC2)^2^ and brain eQTL data^22^ using *Sherlock*^23^ and identified 15 potential causal genes whose expression level change may contribute to schizophrenia risk (Bonferroni corrected *P* < 0.05) (Supplementary Table S3). Of note, *Sherlock* uses collective information from both *cis* (located within 1 Mb of the transcription start site of a gene) and *trans* variants to make statistical inference. Comparing with *cis* variants, the regulatory effects of *trans* variants are relatively difficult to investigate. We thus focused on genes supported by *cis* variants in this study. Among the 15 significant genes, five candidate causal genes were prioritized mainly by *cis* genetic variants (i.e., *cis* variants of these genes showed significant association with schizophrenia and gene expression simultaneously), including *ALMS1*, *GLT8D1*, *ZNF323*, *CSNK2B*, and *TBC1D15*. These five genes therefore represent the most likely causal genes for schizophrenia.

### Causal genes identified by SMR

Through integrating SNP associations from schizophrenia GWAS (PGC2)^2^ and brain eQTL data^22^, *SMR* identified two genes (*SULT2B1* and *ALMS1*) at *P* < 1.0 × 10^−3^ (Supplementary Table S4). However, as *SULT2B1* did not pass *HEIDI* test (*P* < 0.05), only *ALMS1* was retained. Intriguingly, *Sherlock* analysis also suggested that *ALMS1* is a causal gene for schizophrenia (Supplementary Table S3). These consistent results strongly suggest that *ALMS1* may represent a promising causal gene for schizophrenia.

### Causal genes identified by Prix Fixe

We predicted schizophrenia causal genes using *Prix Fixe*, which utilizes functionally coherent subnetworks to prioritize causal genes. In total, 119 genes (PF score > 0) were prioritized by *Prix Fixe*. As our goal is to identify the most possible causal genes, we only retained genes with PF score > 0.10. We found that 41 genes have a PF score > 0.10 (Supplementary Table S5). Of note, *DRD2* ranked the highest among these genes (Supplementary Table S5). Genes ranked from the second to tenth are as follows: *CACNA1C, CACNB2, GRIN2A, CNKSR2, SERPING1, ZNF536, GPM6A, VRK2*, and *GRIA1*. Dysregulation of the dopamine system in the pathophysiology of schizophrenia has been well characterized. In fact, most of antipsychotic drugs exert their effect through blocking dopamine receptors. Thus, the highest PF score of *DRD2* strongly suggests the genes prioritized by *Prix Fixe* may represent promising causal genes for schizophrenia. GO analysis showed that the prioritized causal genes were enriched in synaptic function, neuronal projection, acetylcholine-gated channel complex, and neuronal calcium signaling related categories (Supplementary Table S6).

### Causal genes prioritized by NetWAS

Through integrating gene-wide associations^2^ (SNP-level associations were converted into gene-level associations^28^) and brain-specific functional interaction network^26^, we performed genome-wide prediction of causal genes using *NetWAS*. As our goal is to prioritize the most likely causal genes, top 50 genes were included in this study as promising candidates (Supplementary Table S7). GO analysis showed that neural development and neural projection categories were enriched in the prioritized causal genes (Supplementary Figure S1).

### Causal genes prioritized by DAPPLE

Through using *DAPPLE*, which utilizes PPI to prioritize potential causal genes at the reported risk loci, we identified 83 candidate causal genes (corrected *P* < 0.01) (Supplementary Table S8). The top prioritized genes include *DUS2L, ATXN7, SETD8, CHRNB4, CTNNA1, PCDHA5, KDM4A, TSSK6, EP300, ACD, PCDHA1, PLAA, GATAD2A* and *PCDHA2*. As genes located within the genome-wide significant loci were used as input, in essence, we distilled the promising causal genes at these risk loci. Gene ontology (GO) analysis showed that the prioritized causal genes were enriched in nervous system development (corrected *P* = 9.9 × 10^−3^), cell–cell adhesion (corrected *P* = 2.2 × 10^−3^) and chromatin organization (corrected *P* = 4.08 × 10^−2^) categories (Supplementary Figure S2).

### The integrated landscape of causal genes in schizophrenia

We utilized different approaches (including *Sherlock, SMR, Prix Fixe, NetWAS, DEPICT* and *DAPPLE*) to prioritize the plausible causal genes for schizophrenia. The overlapping genes represent promising plausible causal genes as they were supported by different methods. We therefore ranked the prioritized causal genes through integrating the results from different prioritization approaches. To obtain the global landscape of plausible causal genes, we also integrated candidate causal genes identified in previous studies. Candidate causal genes from other studies are as follows: (1) causal genes prioritized by Pavlides et al.^51^. By integrating blood eQTL data^52^ and GWAS data of schizophrenia (PGC2)^2^, Pavlides et al. prioritized potential causal genes using *SMR*^24^. A total of 17 genes (corrected *P* < 0.05) were included (Supplementary Table S9). (2) Causal genes predicted by Zhu et al.^24^. Through integrating brain eQTL data (The Brain eQTL Almanac (Braineac): http://www.braineac.org/) (brain tissues from a total of 134 individuals)^53^ and GWAS data of schizophrenia (PGC2)^2^, Zhu et al. used *SMR* to prioritize causal genes and identified two candidates, *SNX19* and *NMRAL1*. (3) Causal genes identified by Fromer et al.^54^. Recently, Fromer et al. conducted a large-scale RNA sequencing using brain tissues from schizophrenia cases and normal controls. They generated a comprehensive eQTL resource (from a total of 467 subjects of European ancestry) and prioritized candidate causal genes through integrating eQTL resource and GWAS data of schizophrenia^2^ (using *Sherlock*). In total, 33 genes (corrected *P* < 0.05) were identified (Supplementary Table S10). (4) Causal genes prioritized by *DEPICT*. Through using *DEPICT*, Pers et al. predicted the potential causal genes for schizophrenia recently. They predicted a total of 62 plausible candidate causal genes for schizophrenia (FDR < 0.05) (Supplementary Table S11).

**Fig. 1:**
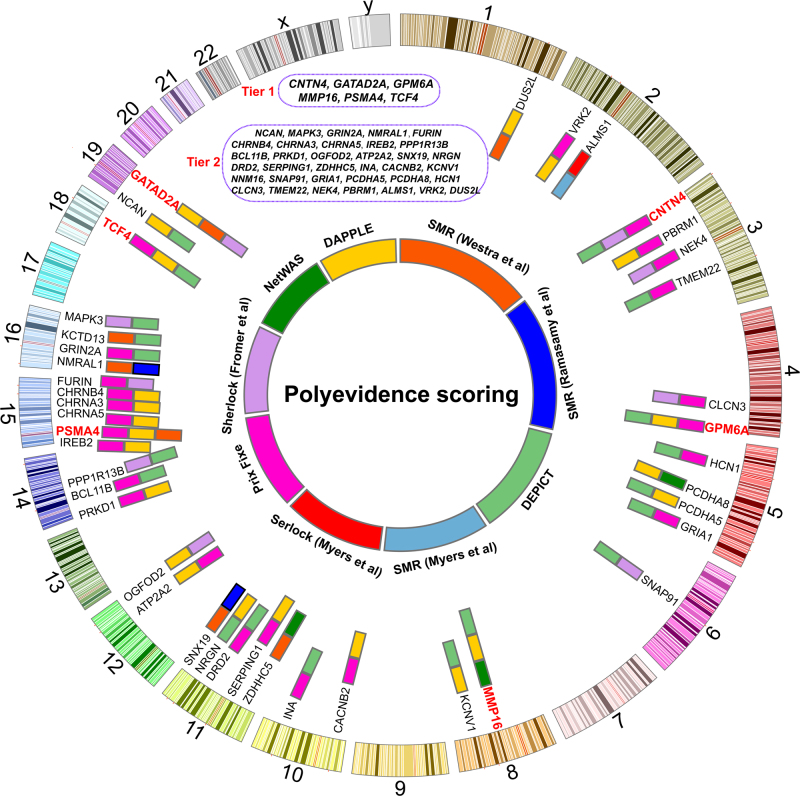
Top causal genes identified in this study. Through integrating the prediction results from different methods, a total of 41 high-confidence causal genes were identified. *CNTN4, GATAD2A, GPM6A, MMP16, PSMA4*, and *TCF4* have the highest scores, these genes therefore represent the most promising causal genes for schizophrenia

On the basis of their frequency of occurrences in the results of different prioritization approaches, we ranked the prioritized causal genes using a cumulative scoring strategy (Materials and methods) and generated the integrated landscape of causal genes in schizophrenia (Fig. 1). A total of 41 promising causal genes (total score > 2 points) were identified through systematically integrating the prediction results from different methods. Six genes (including *CNTN4, GATAD2A, GPM6A, MMP16, PSMA4*, and *TCF4*) have the highest total scores (i.e., 3 points), indicating that at least three different prediction approaches support these genes as plausible causal genes. These six genes thus represent the most promising causal genes for schizophrenia. We therefore called these six genes tier 1 causal genes. In addition, thirty-five genes have a total score of 2 points (these genes were called as tier 2 causal genes). We called these 41 genes high-confidence plausible causal genes as these genes were supported by at least two different prioritization methods.

**Fig. 2:**
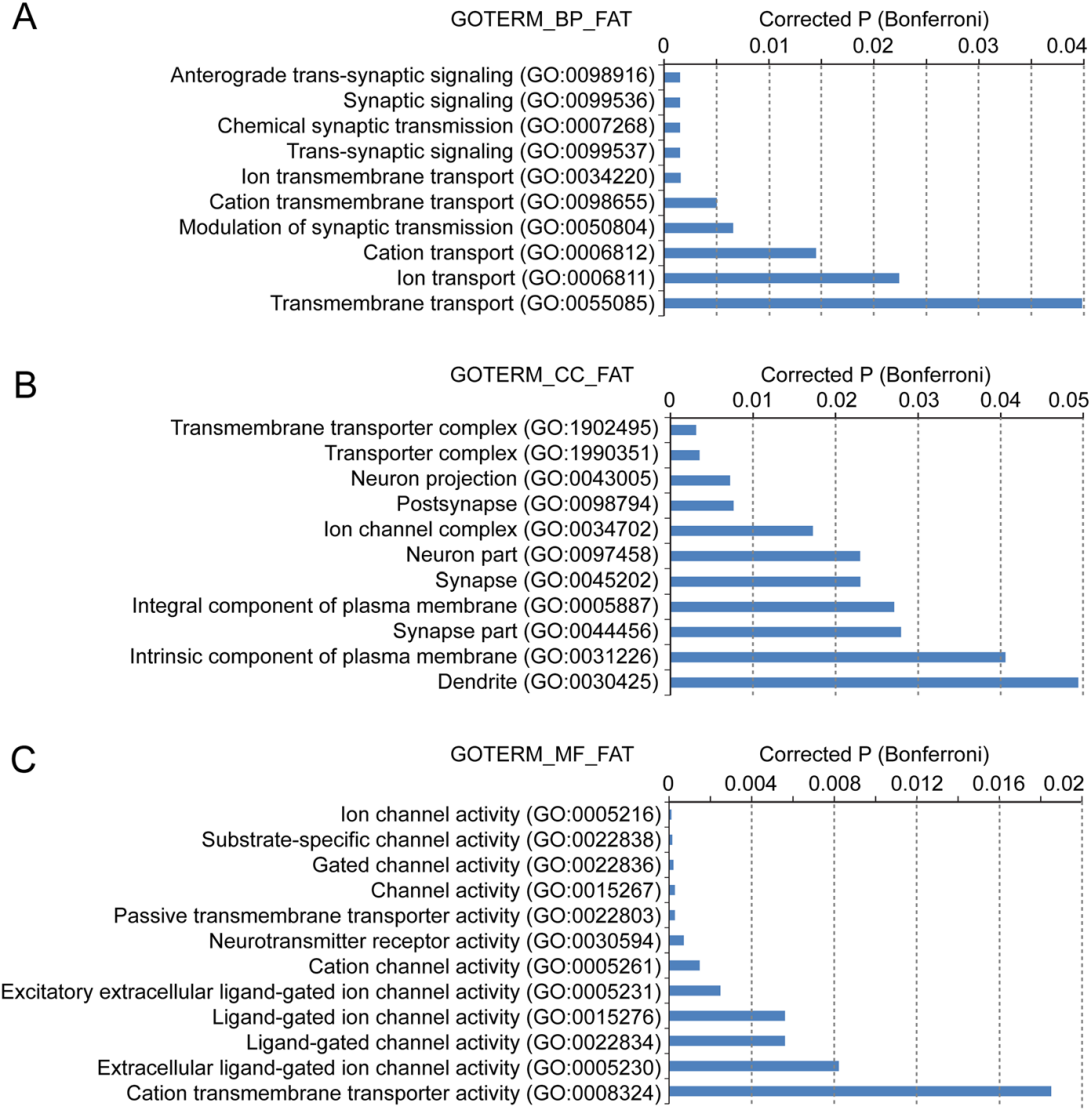
Gene ontology analysis showed that the prioritized causal genes are enriched in synaptic transmission process. Results from different categories were showed, including biological process (**a**), cell component (**b**), and molecular function (**c**)

We performed GO analysis and found that “synaptic transmission” and “ion transport” categories are significantly enriched in the 41 causal genes (Fig. 2a), suggesting that synaptic dysfunction may play a key role in schizophrenia pathogenesis. “Transporter complex”, “neuron projection”, and “postsynapse” categories were significantly enriched among the causal genes when cell component (CC) was used as keyword (Fig. 2b). And “ion channel activity” category was highly significantly overrepresented among the causal genes when molecular function (MF) was used as keyword (Fig. 2b). Taken together, these results suggest that synaptic dysfunction has a critical role in the pathophysiology of schizophrenia.

### Causal genes showed distinct expression pattern in developing human brain

**Fig. 3:**
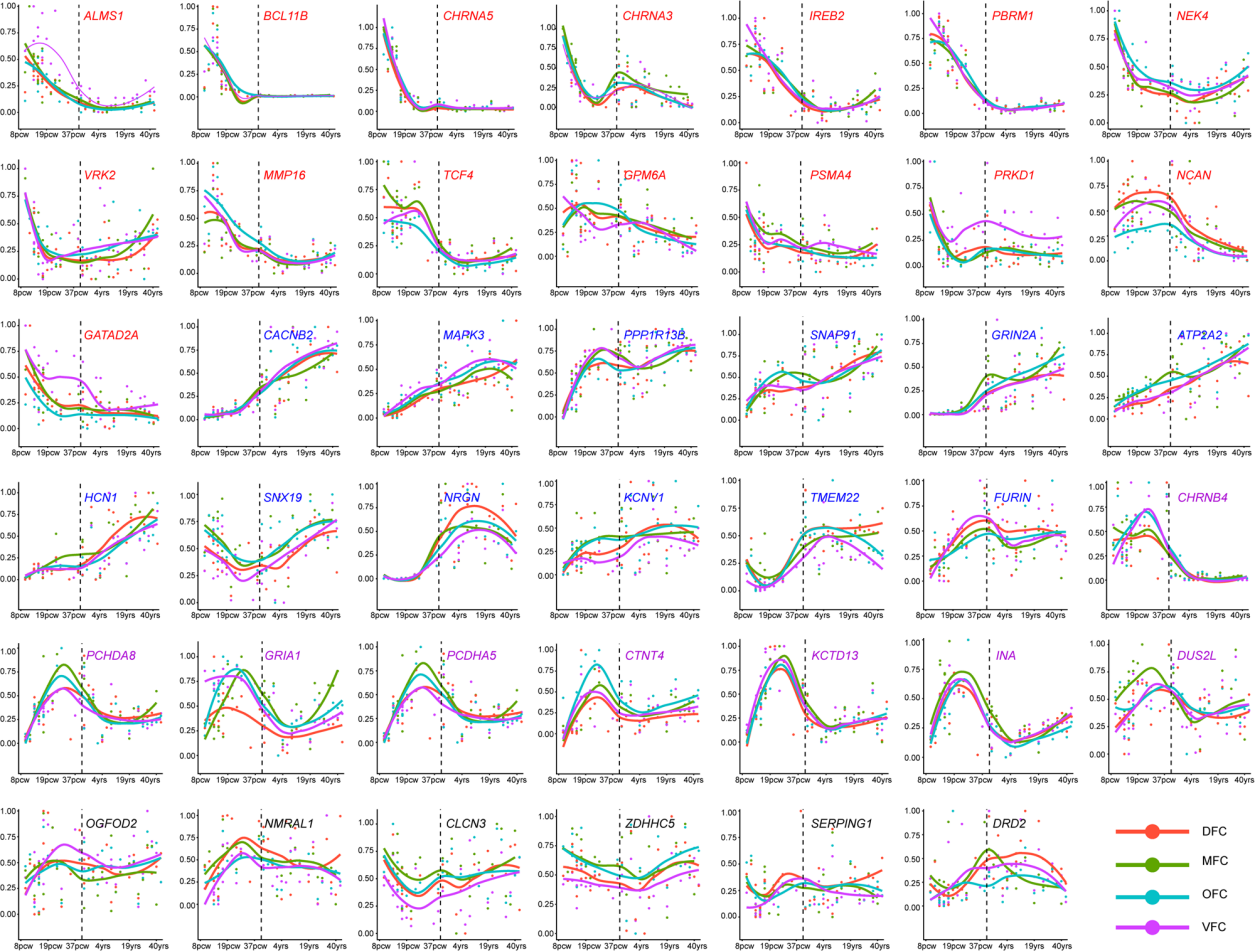
Top causal genes showed distinct expression pattern in developing and adult human brain. Top causal genes were classified into four categories according to their expression pattern. Genes in the first category (marked by red) are highly expressed at early fetal stage, decline at late fetal stage, then maintain a relative stable level from early infancy to adulthood. Expression level of genes in the second category (marked by blue) gradually increases from early fetal stage to adulthood (e.g., *CACNB2* and *MAPK3*). Expression level of genes in category three (marked by pink) gradually increases from early fetal stage to early mid-fetal stage and peaks at late fetal stage. Expression level of genes in category four (marked by black) is relatively stable at different developing stages

We explored the expression of the 41 high-confidence plausible causal genes in developing human brain and found distinct expression patterns in the prefrontal cortex (Fig. 3). We divided these 41 genes into four classes based on their expression trajectory. Expression levels of genes (e.g., *BCL11B* and *CHRNA5*) in the first class (marked by red) are high at early fetal stage, decline at late fetal stage, and then maintain a relative stable level from early infancy to adulthood. In the second class (marked by blue), expression levels of genes gradually increase from early fetal stage to adulthood (e.g., *CACNB2* and *MAPK3*). In the third class (marked by pink), expression levels of genes (e.g., *CHRNB4* and *PCGH8*) gradually increase from early fetal stage to early mid-fetal stage, peaks at late fetal stage, and then decline gradually. Expression levels of genes in the fourth class are relatively stable across entire developing stages (e.g., *ZDHHC5*). These expression patterns suggest that these genes may have different roles at different developmental stages.

### Causal genes are widely expressed in different cell types of CNS

**Fig. 4:**
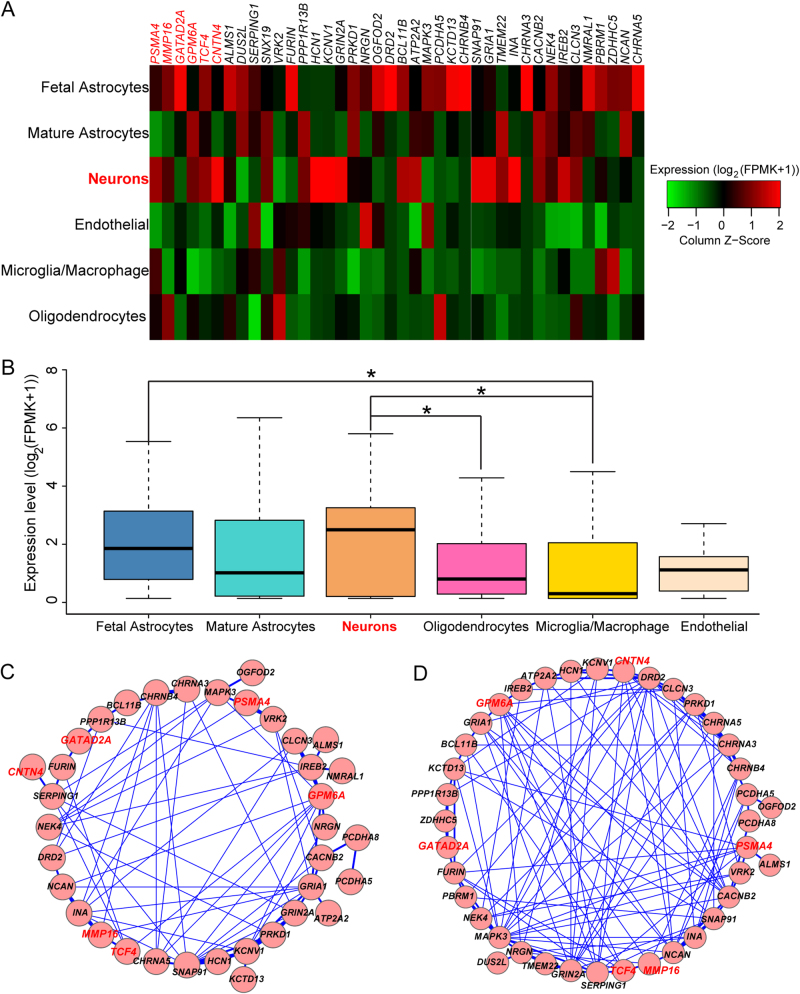
Expression of top causal genes in different cell types. **a** Heatmap showed that the top causal genes are highly expressed in neurons and fetal astrocytes. **b** Expression level of top causal genes in neurons is significantly higher than other cell types. **c** Top causal genes encode a densely interconnected PPI network. **d** Top causal genes form a densely interconnected functional network. *P < 0.05, one-way ANOVA

We analyzed the expression of the prioritized causal genes in different cell types of human brain and found that the 41 prioritized causal genes were highly expressed in fetal astrocytes and neurons (Fig. 4a). The average expression level of the prioritized causal genes was higher in neurons compared with other examined cell types (Fig. 4b), suggesting that these genes are mainly involved in neuronal function. Statistical analysis showed that the average expression level of the predicted causal genes was significantly higher in neurons compared with oligodendrocytes and microglia (*P* < 0.05). In addition, we found that the average expression level of the prioritized causal genes was significantly higher in fetal astrocytes compared with microglia (*P* < 0.05) (Fig. 4b). We also explored the expression of the prioritized causal genes in human ESCs and NSCs derived from ESCs. Again, we found that the 41 high-confidence causal genes were widely expressed in human ESCs and NSCs (Supplementary Figure S3). Of note, compared with tier 2 causal genes, expression level of tier 1 causal genes (i.e., the six genes with the highest total score) are higher in ESCs and NSCs compared with tier 2 causal genes (Supplementary Figure S3). Collectively, these expression data showed that the prioritized causal genes are abundantly expressed in NSCs, neurons and fetal astrocytes, suggesting that these genes may have pivotal roles in CNS.

### Causal genes encode a densely interconnected molecular network

Genes usually act synergistically to exert their biological function in cells, and numerous studies have shown that physically interacted genes are more likely to share function, a phenomenon called guilt-by-association^34,55,56^. We performed network analysis and found that the prioritized causal genes encode a highly interconnected PPI network (Fig. 4c), suggesting these genes may act synergistically in human brain. Using the shared-function (or co-function) network from Tasan et al.^25^, we further found that the prioritized causal genes form a densely interconnected functional network (Fig. 4d). These results suggest that the prioritized causal genes are likely to share biological function. Dysregulation of any member in this molecular network may lead to similar functional consequences (i.e., increase schizophrenia risk).

### Dysregulation of top causal genes in schizophrenia

**Fig. 5:**
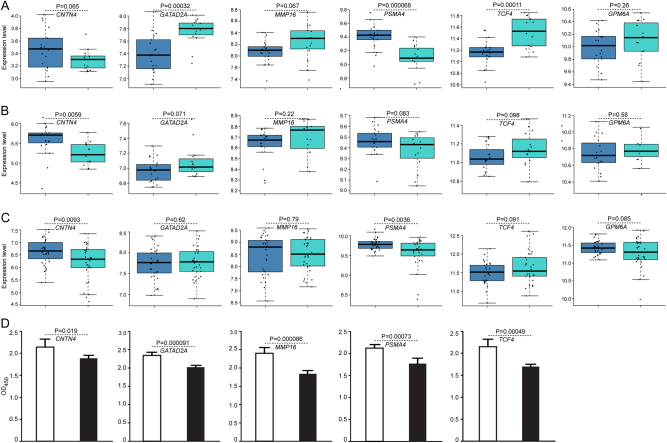
Dysregulation of top causal genes in schizophrenia. **a** Top causal genes were dysregulated in the hippocampus of schizophrenia cases compared with controls. *GATAD2A* and *TCF4* were significantly upregulated in schizophrenia cases, while *PSMA4* was significantly downregulated (*P* < 0.05). Data from GSE53987, which contains 15 schizophrenia cases and 19 controls. **b***CNTN4* was significantly downregulated in the prefrontal cortex of schizophrenia cases. *GATAD2A, PSMA4*, and *TCF4* also showed a trend of dysregulation (*P* < 0.10). Data from GSE53987. (**c**) *CNTN4* and *PSMA4* were significantly downregulated in the prefrontal cortex of schizophrenia cases. *GPM6A* and *TCF4* also showed a trend of dysregulation (*P* < 0.10). Data from GSE21138, which contains 30 schizophrenia cases and 29 controls. **d** Knockdown of *CNTN4, GATAD2A, MMP16, PSMA4*, and *TCF4* impaired proliferation of SH-SY5Y cells

We identified six top causal genes (*CNTN4, GATAD2A, GPM6A, MMP16, PSMA4*, and *TCF4*) through integrating the prediction results from different methods. To further validate the role of these top causal genes in schizophrenia, we compared the expression level of these genes in schizophrenia cases and healthy controls. We found that *GATAD2A* and *TCF4* were significantly upregulated in the hippocampus of schizophrenia cases compared with controls in GSE53987 dataset (*P* < 0.01) (Fig. 5a). In contrast, *PSMA4* was significantly downregulated and *CNTN4* showed a trend of downregulation (*P* = 0.065) in the hippocampus of schizophrenia cases compared with controls (Fig. 5a). In the prefrontal cortex, *CNTN4* was significantly downregulated in schizophrenia cases in both GSE53987 (*P* < 0.01) and GSE21138 (*P* < 0.01) datasets (Fig. 5b, c). Consistent with the downregulation in the hippocampus, *PSMA4* was significantly downregulated in the prefrontal cortex in schizophrenia cases in GSE21138 dataset (*P* < 0.01) (Fig. 5c). Of note, though it did not reach significance level, *TCF4* showed a trend of upregulation in the prefrontal cortex in both GSE53987 and GSE21138 datasets (Fig. 5b, c). Taken together, these results indicate that the top causal genes were dysregulated in schizophrenia cases, supporting that these genes may represent authentic causal genes for schizophrenia.

### Knockdown of the top causal genes impaired the proliferation of SH-SY5Y cells

Expression analysis showed that the top causal genes were dysregulated in schizophrenia cases (Fig. 5). To explore whether the dysregulation of these top causal genes affects cell proliferation, we transiently knocked down these genes in SH-SY5Y neuroblastoma cells. Reverse transcription PCR (RT-PCR) showed that *CNTN4, GATAD2A, MMP16, PSMA4*, and *TCF4* were expressed in SH-SY5Y cells (Supplementary Figure S4). However, the expression of *GMP6A* was not detected. We thus knocked down the expression of *CNTN4, GATAD2A, MMP16, PSMA4*, and *TCF4* using shRNAs and conducted proliferation assays. We found that the shRNAs significantly downregulated the expression of *CNTN4, GATAD2A, MMP16, PSMA4*, and *TCF4* genes (Supplementary Figure S5). Interestingly, knockdown of these top causal genes significantly impaired the proliferation of SH-SY5Y cells (Fig. 5d). Of note, previous study also showed that knockdown of *TCF4* attenuated the proliferation of cortical progenitor cells. Collectively, these results indicate that dysregulation of top causal genes affects proliferation of neuronal cells, suggesting these genes may play a role in neurodevelopment.

## Discussion

Recent GWAS have identified numerous schizophrenia risk loci. However, it is difficult to pinpoint the causal gene as each risk locus usually contains multiple highly linked genetic variants. In addition, gene regulatory is complex. For example, recent studies have showed that genetic variant may regulate the activity of distal gene through long-range chromatin interactions^20,57^. Thus, in many cases, the gene (s) nearest to the most significant genetic variant (identified by GWAS) may not represent the authentic causal gene (s). Compared with the rapid discovery of schizophrenia risk variants and loci, the identification of causal genes lags far behind. In this study, we systematically predicted the causal genes for schizophrenia by utilizing several well-characterized methods. Through integrating the results from different approaches, we prioritized 41 high-confidence causal genes for schizophrenia. Of note, a recent study also identified *GATAD2A, PSMA4, FURIN*, and *OGFOD2* as schizophrenia risk genes through integrating genetic associations from GWAS and eQTL data from diverse tissues^58^, further supporting the notation that these genes may have a role in schizophrenia.

Among the 41 prioritized causal genes, *CNTN4, GATAD2A, GPM6A, MMP16, PSMA4*, and *TCF4* represent the most promising causal genes for schizophrenia. *CNTN4* encodes contactin-4 protein, a member of the immunoglobulin superfamily. As a neuronal adhesion molecule, contactin-4 plays a role in developing nervous system through influencing axon guidance and fasciculation^59,60^. In addition to PGC2, previous studies have showed that *CNTN4* is associated with schizophrenia^61^ and disruption of *CNTN4* caused developmental delay^62^. A recent study also showed that dysregulation of *CNTN4* impaired proliferation of neural progenitor cells^54^. Interestingly, several groups reported that *CNTN4* is associated with autism^63,64^, a neurodevelopmental disorder. These results strongly suggest that *CNTN4* may play a role in schizophrenia through influencing neurodevelopment. The function of *GATAD2A* remains largely unknown. However, recent studies showed that *GATAD2A* is associated with diabetes^65^ and several types of cancer (breast, ovarian, and prostate cancer)^66^. Consistent with our observation, Wang et al showed that knockdown of *GATAD2A* suppressed cell proliferation in thyroid cancer^67^ and Marino showed that loss of *GATAD2A* function in mice is embryonic lethal. These results suggest that *GATAD2A* plays a crucial role in development. Glycoprotein M6A (*GPM6A*) encodes a transmembrane protein that is abundantly expressed in cell surface of neurons in the CNS^68^. Multiple studies have showed that *GPM6A* plays a pivotal role in neurodevelopment through regulating neuronal migration, differentiation, neurite outgrowth, spine formation, and synaptogenesis^69–72^. Intriguingly, a recent study showed that altered *GPM6A* dosage impairs cognition^73^, a phenotype that is frequently reported to be impaired in schizophrenia. These results strongly suggest that *GPM6A* may play a role in schizophrenia by affecting brain development. *MMP16* encodes matrix mentalloproteinase-16, an enzyme that is response for breakdown of extracellular matrix^74^. Previous studies have shown that MMPs are involved in invasion^75^ and migration of cancer cells^76,77^. *PSMA4* encodes proteasome subunit alpha type-4^78^, a member of the 20S proteasome complex. GWAS showed that *PSMA4* is associated with lung cancer^79^ and smoking^80^. Of note, Han et al. showed that PSMA4 protein is interacted with DTNBP1, a protein encoded by a promising schizophrenia candidate gene^81^. *TCF4* encodes transcription factor 4, a pivotal transcription factor that plays critical role in development. *TCF4* is one of the most frequently reported schizophrenia risk genes. Numerous GWASs repeatedly reported the association of *TCF4* with schizophrenia^2,15,82^, strongly suggesting that *TCF4* is a causal gene for schizophrenia. Accumulating evidence supports that *TCF4* plays pivotal role in neurodevelopment through regulating the columnar distribution of lay2/3 prefrontal pyramidal neurons^83^, synaptic plasticity and memory function^84^. Recent studies also showed that *TCF4* is associated with cognitive functions in mouse and humans^85,86^. These lines of evidence suggest that *TCF4* may have a crucial role in schizophrenia pathogenesis by modulating neurodevelopment.

The top 41 predicted causal genes are highly expressed in neurons (Fig. 4a, b) and are enriched in synaptic transmission-related pathways (Fig. 2), suggesting these predicted causal genes exert their main functions in the central nervous system (CNS). However, we noticed that several genes (including *ATP2A2, PSMA4, PBRM1, SERPING1*, and *VRK2*) were also highly expressed in microglia (Fig. 4a), the resident macrophage cells that act as the first and main form of active immune defense in the CNS^87^. The high expression level of these genes in microglia implies that these genes may also have a role in neuro-immunity through regulating the function of microglia. Nevertheless, more work is needed to elucidate the role of these genes in neuro-immunity.

There are several limitations of this study. First, only genes supported by at least two different prioritization methods were selected in this study. Though these genes are promising causal genes for schizophrenia, genes supported by individual prediction approach may also have a role in schizophrenia. Second, the prioritization methods used data from PGC2 as a primary source to predict the causal genes for schizophrenia, validation of these genes in independent schizophrenia samples will provide direct support for the involvement of these gene in schizophrenia. Third, though this study identified promising causal genes, further biological experiments are needed to demonstrate the role of the prioritized genes in schizophrenia. Fourth, the number of eQTL datasets used in this study is relatively limited as only three brain eQTL datasets and one blood eQTL dataset^22,52–54^ were included in this study. Accordingly, the number of potential causal genes identified from the four eQTL datasets might be relatively small. Of note, Hauberg et al. integrated large-scale GWAS and multiple eQTL datasets recently and they identified numerous disease-associated genes^58^. Interestingly, Hauberg et al. showed that eQTLs derived from pathophysiologically relevant tissues play a pivotal role in the identification of disease-associated risk genes. As schizophrenia is a mental disease that mainly originates from abnormal brain function, brain eQTL is more suitable than eQTLs from other tissues. Thus, risk genes identified using brain eQTLs may represent promising candidate genes for schizophrenia. More importantly, as we used lines of convergent evidence to predict the causal genes for schizophrenia, we utilized other prediction methods (such as *Prix Fixe* and *DEPICT*) and provided further evidence that support the risk genes identified by integrative analysis (i.e., integrating GWAS signals and eQTLs).

We generated the landscape of causal genes in schizophrenia for the first time. In essence, we distilled the findings of GWAS of schizophrenia. Thus, the identified genes represent the most promising causal genes for schizophrenia. In fact, all of the top six causal genes showed significant association with schizophrenia at genome-wide level (Supplementary Figure S6-S11). GO analysis further showed that identified causal genes were enriched in synaptic signaling pathway, further supporting the notion that synaptic dysregulation may have a key role in schizophrenia. Of note, five (*CNTN4, GATAD2A, GPM6A, MMP16*, and *TCF4*) of the top six causal genes are involved in neurodevelopment, further supporting the neurodevelopmental hypothesis of schizophrenia.

## Electronic supplementary material

SUPPLEMENTAL MATERIA(DOCX 1968 kb)
