# Supplementary material for: The integrated landscape of causal genes and pathways in schizophrenia
Source: Transl Psychiatry. 2018 Mar 15;8:67. doi: 10.1038/s41398-018-0114-x (PMC5851982; doi:10.1038/s41398-018-0114-x)
Supplement: Supplementary file 1 — SUPPLEMENTAL MATERIA(DOCX 1968 kb) [file 41398_2018_114_MOESM1_ESM.docx]

**The integrated landscape of causal genes and pathways in schizophrenia**

Changguo Ma^1^, Chunjie Gu^1^, Yongxia Huo^1^, Xiaoyan Li^1^, Xiong-Jian Luo^1,*^

^1^Key Laboratory of Animal Models and Human Disease Mechanisms of the Chinese Academy of Sciences & Yunnan Province, Kunming Institute of Zoology, Chinese Academy of Sciences, Kunming, Yunnan 650223, China

*To whom correspondence should be addressed; Key Laboratory of Animal Models and Human Disease Mechanisms, Kunming Institute of Zoology, Chinese Academy of Sciences, Kunming, Yunnan 650223, China; Tel: +86-871-68125413, Fax: +86-871-68125413, E-mail: [luoxiongjian@mail.kiz.ac.cn](mailto:luoxiongjian@mail.kiz.ac.cn).

**Supplementary Methods**

***SMR integrative analysis***

*Sherlock* uses Bayesian inference while *SMR* utilizes Mendelian Randomization. Briefly, if a genetic variant (e.g., SNP X) is associated with gene expression (e.g., gene Y), individuals with different genotypes at SNP X will have different expression level of Y. Thus, if the expression change of gene Y has an effect on a trait (e.g., disease risk), phenotype differences will be observed among subjects with different genotypes at SNP X (i.e., the SNP X will also have an effect on the trait). This method is similar to Mendelian randomi­zation (*MR*) analysis. In *MR*, researchers usually utilize a genetic variant as instrumental variable to predict the potential effect of an exposure (e.g., expression) on an outcome (trait or phenotype). Thus, theoretically, *MR* analysis can be used to predict the most plausible functional genes at the loci identified by GWAS.

***Brain eQTL data***

Gene regulation has strong tissue and cell-type specificity^1^. That is, a large proportion of genetic variants only exerts their regulatory effect in some specific tissues. As a matter of fact, Ripke et al. showed that schizophrenia association signals were enriched at enhancers that are active in human brain and immune-related tissues^2^, providing further evidence that supports the tissue-specific regulation effects of schizophrenia-associated genetic variants. Though the genetic and pathophysiological mechanisms of schizophrenia remains largely unknown, accumulating evidence suggests that schizophrenia is caused by abnormal neurodevelopment^3, 4^. Therefore, human brain may represent an ideal tissue to investigate the regulation effects of schizophrenia-associated genetic variation. We thus used brain eQTL from Myer et al in this study^5^.

***Quantitative Real-time PCR***

Knockdown efficiency was measured by quantitative real-time PCR (qPCR). SH-SY5Y cells were transfected with shRNA constructs and cultured for 48 hours, and total RNA was then extracted with TRIzol (ambion) reagent. First-strand cDNA was synthesized using the FastQuant reverse transcriptase kit (TIANGEN). Quantitative PCR was performed using Bio-Rad CFX96 detection system using the SYBR Green kit (Bio-Rad). The sequences for qPCR primers used are listed in Table S1. Actin was used as internal controls and the relative change in expression was calculated using the 2^-△△Ct^ method.

**Supplementary Tables and Figures**

**Table S1. The sequences of the qPCR primers**

| **Gene** | **Forward primer (5’→3’)** | **Reverse primer (5’→3’)** |
| --- | --- | --- |
| *CNTN4* | GCCAAAAGCGTCTCCAAAACC | ACTCCCAGCGTCTGATTTAGTAA |
| *GATAD2A* | GAGCCCGAGAGTGAATGGG | TTCAACAACACGAGTTTTGCTTC |
| *MMP16* | ATGCAGCAGTTCTATGGCATT | CTGGTCAGGTACACCGCATC |
| *PSMA4* | AGTGTGGCAGGCATAACTTCT | TCACAAGGTATTGGCTCCTGA |
| *TCF4* | CAAGCACTGCCGACTACAATA | CCAGGCTGATTCATCCCACTG |
| β-Actin | CATGTACGTTGCTATCCAGGC | CTCCTTAATGTCACGCACGAT |

**Table S2. The sequences of shRNA oligonucleotides**

| **Gene** | **Sequence (5’→3’)** |
| --- | --- |
| *Control* | CCTTGCTATTTCAACTTCCTT |
| *CNTN4* | GCTTCAGTTTGCTTATCTTGA |
| *GATAD2A* | GCACCTCACACAGTGACATGA |
| *MMP16* | GCAGCAGTTCTATGGCATTAA |
| *PSMA4* | GGGATAAGCACTATGGCTTTC |
| *TCF4* | GGGATAAGCACTATGGCTTTC |

**Table S3. Causal genes identified by *Sherlock***

| **Gene symbol** | **LBF^a^** | ***P*-value^b^** | **Supporting SNP**  **(*cis* or *trans*)^c^** | ***P*_eQTL_^d^** | ***P*_GWAS_^e^** | ***P_corrected_^f^*** |
| --- | --- | --- | --- | --- | --- | --- |
| [***LOC376138***](http://www.genecards.org/cgi-bin/carddisp.pl?gene=LOC376138) | 9.38 | 1.11×10^-6^ | [rs4898494](http://www.ncbi.nlm.nih.gov/projects/SNP/snp_ref.cgi?rs=4898494) (*trans*) | 3.08×10^-17^ | 7.04×10^-6^ | <0.05 |
|  |  |  | [rs902302](http://www.ncbi.nlm.nih.gov/projects/SNP/snp_ref.cgi?rs=902302) (*trans*) | 7.95×10^-7^ | 3.82×10^-5^ |  |
| [***KDELR1***](http://www.genecards.org/cgi-bin/carddisp.pl?gene=KDELR1) | 8.82 | 1.11×10^-6^ | [rs4735158](http://www.ncbi.nlm.nih.gov/projects/SNP/snp_ref.cgi?rs=4735158) (*trans*) | 8.04×10^-6^ | 3.33×10^-9^ |  |
|  |  |  | [rs195204](http://www.ncbi.nlm.nih.gov/projects/SNP/snp_ref.cgi?rs=195204) (*trans*) | 1.85×10^-6^ | 2.48×10^-4^ | <0.05 |
|  |  |  | [rs16954069](http://www.ncbi.nlm.nih.gov/projects/SNP/snp_ref.cgi?rs=16954069) (*trans*) | 9.22×10^-6^ | 8.33×10^-4^ |  |
| [***ALMS1***](http://www.genecards.org/cgi-bin/carddisp.pl?gene=ALMS1) | 7.63 | 1.11×10^-6^ | [rs6753344](http://www.ncbi.nlm.nih.gov/projects/SNP/snp_ref.cgi?rs=6753344) (*cis*)  rs11191393(*trans*) | 1.37×10^-9^  4.62×10^-6^ | 1.77×10^-6^  1.15×10^-3^ | <0.05 |
| [***GLT8D1***](http://www.genecards.org/cgi-bin/carddisp.pl?gene=GLT8D1) | 7.10 | 1.11×10^-6^ | [rs6795646](http://www.ncbi.nlm.nih.gov/projects/SNP/snp_ref.cgi?rs=6795646) (*cis*) | 2.50×10^-8^ | 7.15×10^-9^ | <0.05 |
| ***ZNF323*** | 7.05 | 1.11×10^-6^ | rs1150709 (*cis*) | 2.05×10^-8^ | 7.05×10^-10^ | <0.05 |
| [***CSNK2B***](http://www.genecards.org/cgi-bin/carddisp.pl?gene=CSNK2B) | 6.70 | 1.11×10^-6^ | [rs2075800](http://www.ncbi.nlm.nih.gov/projects/SNP/snp_ref.cgi?rs=2075800) (*cis*) | 5.39×10^-6^ | 3.63×10^-11^ | <0.05 |
| [***SLITRK5***](http://www.genecards.org/cgi-bin/carddisp.pl?gene=SLITRK5) | 6.16 | 2.21×10^-6^ | [rs1265883](http://www.ncbi.nlm.nih.gov/projects/SNP/snp_ref.cgi?rs=1265883) (*trans*)  [rs6465984](http://www.ncbi.nlm.nih.gov/projects/SNP/snp_ref.cgi?rs=6465984)(*trans*) | 7.49×10^-6^  4.48×10^-6^ | 5.04×10^-5^  1.41×10^-4^ | <0.05 |
| [***LOC375768***](http://www.genecards.org/cgi-bin/carddisp.pl?gene=LOC375768) | 5.93 | 2.21×10^-6^ | [rs9853627](http://www.ncbi.nlm.nih.gov/projects/SNP/snp_ref.cgi?rs=9853627) (*trans*) | 4.85×10^-7^ | 1.09×10^-6^ | <0.05 |
| [***TBC1D15***](http://www.genecards.org/cgi-bin/carddisp.pl?gene=TBC1D15) | 5.65 | 4.42×10^-6^ | [rs6582065](http://www.ncbi.nlm.nih.gov/projects/SNP/snp_ref.cgi?rs=6582065) (*cis*) | 4.96×10^-6^ | 5.12×10^-5^ | <0.05 |
| [***ZNF446***](http://www.genecards.org/cgi-bin/carddisp.pl?gene=ZNF446) | 5.64 | 4.42×10^-6^ | [rs7012579](http://www.ncbi.nlm.nih.gov/projects/SNP/snp_ref.cgi?rs=7012579) (*trans*) | 4.38×10^-7^ | 1.51×10^-5^ | <0.05 |
|  |  |  | [rs413019](http://www.ncbi.nlm.nih.gov/projects/SNP/snp_ref.cgi?rs=413019)(*trans*) | 8.04×10^-6^ | 2.67×10^-4^ |  |
| ***RCN1*** | 5.46 | 4.42×10^-6^ | rs3800917 (*trans*) | 6.56×10^-7^ | 3.86×10^-10^ | <0.05 |
| [***ADAM15***](http://www.genecards.org/cgi-bin/carddisp.pl?gene=ADAM15) | 5.43 | 4.42×10^-6^ | [rs17073903](http://www.ncbi.nlm.nih.gov/projects/SNP/snp_ref.cgi?rs=17073903) (*trans*) | 1.89×10^-6^ | 2.17×10^-7^ | <0.05 |
| [***UBE2O***](http://www.genecards.org/cgi-bin/carddisp.pl?gene=UBE2O) | 5.43 | 4.42×10^-6^ | [rs4976976](http://www.ncbi.nlm.nih.gov/projects/SNP/snp_ref.cgi?rs=4976976)(*trans*) | 7.14×10^-6^ | 1.19×10^-10^ | <0.05 |
|  |  |  | [rs17078997](http://www.ncbi.nlm.nih.gov/projects/SNP/snp_ref.cgi?rs=17078997)(*trans*) | 6.59×10^-6^ | 1.40×10^-3^ |  |
| [***PAAF1***](http://www.genecards.org/cgi-bin/carddisp.pl?gene=PAAF1) | 5.32 | 4.42×10^-6^ | [rs1471568](http://www.ncbi.nlm.nih.gov/projects/SNP/snp_ref.cgi?rs=1471568) (*trans*) | 4.63×10^-7^ | 4.65×10^-4^ | <0.05 |
|  |  |  | [rs12512143](http://www.ncbi.nlm.nih.gov/projects/SNP/snp_ref.cgi?rs=12512143) (*trans*) | 1.01×10^-6^ | 2.96×10^-3^ |  |
| [***MEGF6***](http://www.genecards.org/cgi-bin/carddisp.pl?gene=MEGF6) | 5.21 | 4.42×10^-6^ | [rs7018304](http://www.ncbi.nlm.nih.gov/projects/SNP/snp_ref.cgi?rs=7018304) (*trans*) | 1.83×10^-6^ | 6.43×10^-9^ | <0.05 |

^a^LBF, Log Bayes Factors, a statistics that reflects if the combined evidence from GWAS and eQTL support a gene being associated with disease. High Bayes factors suggests high probability that the gene is associated with schizophrenia. For a given gene, individual LBF was calculated for each SNP and the sum of these constitutes the final LBF score for the gene. ^b^*P*-value from *Sherlock* integrative analysis. ^c^SNPs were defined as *cis* if they are located within 1 Mb of the transcription start site of a gene. ^d^P value from brain eQTL (Myers et al^5^.). ^e^P from schizophrenia GWAS (PGC2)^2^. Genes with LBF>5.0 are listed. ^f^Bonferroni corrected P value. Genes supported by *cis* genetic variants were marked by red.

**Table S4. Causal genes identified by *SMR***

| **Gene** | **Probe_bp** | **SNP** | **SNP_bp** | **p_GWAS** | **p_eQTL** | **p_SMR** | **p_HET^a^** |
| --- | --- | --- | --- | --- | --- | --- | --- |
| *SULT2B1* | 53747241 | r12104272 | 54839864 | 1.57×10^-5^ | 1.33×10^-21^ | 8.55×10^-5^ | 6.25×10^-5^ |
| ***ALMS1*** | 73466394 | r7607892 | 73730362 | 3.19×10^-6^ | 1.54×10^-9^ | 2.22×10^-4^ | 5.18×10^-1^ |

^a^*P* value of HEIDI test^6^, only genes with p_HET>0.05 were retained as plausible causal genes.

**Table S5. Causal genes identified by *Prix Fixe***

| Symbol | Chromosome | Region | PF Score^a^ |
| --- | --- | --- | --- |
| *DRD2* | chr11 | q23.2 | 0.30182 |
| *CACNA1C* | chr12 | p13.33 | 0.26142 |
| *CACNB2* | chr10 | p12.31--p12.33 | 0.26122 |
| *GRIN2A* | chr16 | p13.2 | 0.24894 |
| *CNKSR2* | chrX | p22.12 | 0.22427 |
| *SERPING1* | chr11 | q12.1 | 0.21958 |
| *ZNF536* | chr19 | q12 | 0.21251 |
| *GPM6A* | chr4 | q34.2 | 0.21157 |
| *VRK2* | chr2 | p16.1 | 0.20498 |
| *GRIA1* | chr5 | q33.2 | 0.19705 |
| *ATP2A2* | chr12 | q24.11 | 0.17027 |
| *FURIN* | chr15 | q26.1 | 0.16632 |
| *CHRNA3* | chr15 | q25.1 | 0.16336 |
| *FES* | chr15 | q26.1 | 0.16322 |
| *PSMA4* | chr15 | q25.1 | 0.15469 |
| *KCNJ13* | chr2 | q37.1 | 0.15314 |
| *SEPT3* | chr22 | q13.2 | 0.15174 |
| *CHRNA5* | chr15 | q25.1 | 0.15018 |
| *PRKD1* | chr14 | q12 | 0.14700 |
| *CEP170* | chr1 | q43 | 0.14572 |
| *CNTN4* | chr3 | p26.2--p26.3 | 0.14537 |
| *STAG1* | chr3 | q22.3 | 0.14228 |
| *HCN1* | chr5 | p12 | 0.14110 |
| *PBRM1* | chr3 | p21.1 | 0.13989 |
| *GNL3* | chr3 | p21.1 | 0.13802 |
| *ADAMTSL3* | chr15 | q25.2 | 0.13707 |
| *CLCN3* | chr4 | q33 | 0.13474 |
| *IREB2* | chr15 | q25.1 | 0.13346 |
| *SMG6* | chr17 | p13.3 | 0.13098 |
| *CLP1* | chr11 | q12.1 | 0.12389 |
| *CHRNB4* | chr15 | q25.1 | 0.12308 |
| *ITIH3* | chr3 | p21.1 | 0.12012 |
| *NEK4* | chr3 | p21.1 | 0.11851 |
| *ETF1* | chr5 | q31.2 | 0.11248 |
| *PTPRF* | chr1 | p34.1--p34.2 | 0.11075 |
| *SFMBT1* | chr3 | p21.1 | 0.11014 |
| *TCF4* | chr18 | q21.2 | 0.10896 |
| *BCL11B* | chr14 | q32.2 | 0.10714 |
| *MAN2A1* | chr5 | q21.3 | 0.10671 |
| *TMEM22* | chr3 | q22.3 | 0.10146 |
| *PSMD6* | chr3 | p14.1 | 0.10060 |

^a^High PF score suggest high probability that the gene is a causal gene.

**Table S6. GO analysis of the causal genes prioritized by *Prix Fixe***

| **GO Category** | **Term** | **Genes** | **P values** | **Corrected P value^a^** |
| --- | --- | --- | --- | --- |
| GOTERM_CC_DIRECT | Postsynaptic membrane | *CHRNA3, CHRNA5, CHRNB4, CNKSR2, GRIA1, GRIN2A* | 8.2×10^-5^ | 9.7×10^-3^ |
| GOTERM_CC_DIRECT | Neuron projection | *BCL11B, CHRNB4, CNKSR2, GRIN2A, GPM6A, PTPRF* | 1.9×10^-4^ | 1.1×10^-2^ |
| GOTERM_CC_DIRECT | Postsynaptic density | *CACNA1C, CHRNA3, DRD2, GRIA1, GRIN2A* | 3.9×10^-4^ | 1.5×10^-2^ |
| GOTERM_CC_DIRECT | Neuronal cell body | *CHRNA3, CHRNA5, CNKSR2, GRIA1, GPM6A, PTPRF* | 9.5×10^-4^ | 2.8×10^-2^ |
| GOTERM_CC_DIRECT | Acetylcholine-gated channel complex | *CHRNA3, CHRNA5, CHRNB4* | 1.2×10^-3^ | 2.8×10^-2^ |
| GOTERM_BP_DIRECT | Calcium ion transmembrane transport | *ATP2A2, CACNB2, CACNA1C, GRIN2A, GPM6A* | 1.5×10^-4^ | 3.1×10^-2^ |

^a^The *P*-values of the over-represented GO terms was corrected by the Benjamini-Hochberg procedure.

**Table S7. Causal genes identified by *NetWAS***

| **Gene** | **Chromosome** | **NetWAS score** |
| --- | --- | --- |
| *DCP2* |  | 2.68306 |
| *FAM216A* |  | 2.64843 |
| *FLRT2* |  | 2.5612 |
| *PIGK* |  | 2.55335 |
| *PFKL* |  | 2.49162 |
| *HIST1H2AM* |  | 2.47332 |
| *ADNP* |  | 2.4648 |
| *UBE2B* |  | 2.42363 |
| *HRC* |  | 2.39457 |
| *RTN4* |  | 2.3919 |
| *HIST1H3F* |  | 2.37396 |
| *PSMD3* |  | 2.35213 |
| *NCAPH2* |  | 2.31398 |
| *SP3* |  | 2.30342 |
| *ZDHHC5* |  | 2.30226 |
| *RNF181* |  | 2.29068 |
| *PCDHA8* |  | 2.27471 |
| *DCUN1D4* |  | 2.26486 |
| *TNIP2* |  | 2.26049 |
| *CHD9* |  | 2.25543 |
| *P4HB* |  | 2.25346 |
| *MLXIP* |  | 2.25022 |
| *RIMS2* |  | 2.24767 |
| *FLRT3* |  | 2.22679 |
| *SLC24A4* |  | 2.224 |
| *RBBP6* |  | 2.22187 |
| *GNB4* |  | 2.22095 |
| *KALRN* |  | 2.21294 |
| *NEO1* |  | 2.20874 |
| *RAB28* |  | 2.20712 |
| *BRD4* |  | 2.1959 |
| *MAGI1* |  | 2.19443 |
| *LYRM1* |  | 2.18974 |
| *SAV1* |  | 2.18438 |
| *DNM1* |  | 2.18321 |
| *HTR2A* |  | 2.17809 |
| *PAX3* |  | 2.17244 |
| *HS3ST2* |  | 2.16948 |
| *BACE1* |  | 2.16664 |
| *SEPHS1* |  | 2.16643 |
| *KATNB1* |  | 2.16438 |
| *CDS1* |  | 2.15504 |
| *KIAA1279* |  | 2.15456 |
| *SDF2* |  | 2.15362 |
| *DNAJB14* |  | 2.12537 |
| *RAB31* |  | 2.12493 |
| *SLIT2* |  | 2.11925 |
| *UCHL1* |  | 2.11045 |
| *EPHB2* |  | 2.10856 |
| *MMP16* |  | 2.10777 |

**Table S8. Causal genes identified by *DAPPLE***

| GENE | P_corrected |
| --- | --- |
| *DUS2L* | 0.001997 |
| *ATXN7* | 0.001997 |
| *SETD8* | 0.001997 |
| *CHRNB4* | 0.001997 |
| *CTNNA1* | 0.001997 |
| *PCDHA5* | 0.001997 |
| *KDM4A* | 0.001997 |
| *TSSK6* | 0.001997 |
| *EP300* | 0.001997 |
| *ACD* | 0.001997 |
| *PCDHA1* | 0.001997 |
| *PLAA* | 0.001997 |
| *GATAD2A* | 0.001997 |
| *PCDHA2* | 0.001997 |
| *OTUD7B* | 0.003992 |
| *GPM6A* | 0.003992 |
| *CTNND1* | 0.003992 |
| *HIST1H2BL* | 0.003992 |
| *HIST1H2BJ* | 0.003992 |
| *PCDHA9* | 0.003992 |
| *PBRM1* | 0.003992 |
| *PCDHA8* | 0.005985 |
| *CHRNA3* | 0.005985 |
| *CENPM* | 0.005985 |
| *PCDHA3* | 0.005985 |
| *NRGN* | 0.005985 |
| *MYO18B* | 0.005985 |
| *INA* | 0.007976 |
| *C12orf65* | 0.007976 |
| *SERPING1* | 0.007976 |
| *HSPD1* | 0.007976 |
| *KCNV1* | 0.009965 |
| *CHRNA5* | 0.009965 |
| *PSKH1* | 0.009965 |
| *PCDHA7* | 0.011952 |
| *TLE1* | 0.011952 |
| *EGR1* | 0.013937 |
| *TCF4* | 0.013937 |
| *INO80E* | 0.013937 |
| *SREBF2* | 0.013937 |
| *PRKD1* | 0.013937 |
| *MMP16* | 0.01592 |
| *SLC12A4* | 0.01592 |
| *NAB2* | 0.01592 |
| *PSMB10* | 0.017901 |
| *CACNB2* | 0.017901 |
| *DFNA5* | 0.01988 |
| *BCL9* | 0.01988 |
| *HARS* | 0.01988 |
| *EPC2* | 0.01988 |
| *HSPE1* | 0.021857 |
| *ACTR5* | 0.021857 |
| *CENPT* | 0.021857 |
| *RRAS* | 0.023832 |
| *HIRIP3* | 0.025805 |
| *PCDHA6* | 0.025805 |
| *OGFOD2* | 0.025805 |
| *BOLL* | 0.027776 |
| *PTN* | 0.029745 |
| *ANP32E* | 0.029745 |
| *ERCC4* | 0.029745 |
| *SHMT2* | 0.031713 |
| *CDC25C* | 0.033678 |
| *LRP1* | 0.037602 |
| *ATP2A2* | 0.037602 |
| *VRK2* | 0.037602 |
| *ZMAT2* | 0.039561 |
| *PSMA4* | 0.039561 |
| *PLEKHO1* | 0.039561 |
| *CD14* | 0.041518 |
| *MAD1L1* | 0.041518 |
| *IREB2* | 0.041518 |
| *ARHGAP1* | 0.043473 |
| *NCAN* | 0.043473 |
| *IK* | 0.043473 |
| *PCDHA4* | 0.045426 |
| *OSBPL3* | 0.045426 |
| *CKB* | 0.045426 |
| *TCF20* | 0.045426 |
| *SREBF1* | 0.047377 |
| *MEF2C* | 0.047377 |
| *NLGN4X* | 0.047377 |
| *ARL3* | 0.047377 |

**Table S9. Causal genes identified by *SMR* (Blood eQTL from Westra et al^7^)**

| **Gene** | **Probe_bp** | **SNP** | **SNP_bp** | **p_GWAS** | **p_eQTL** | **p_SMR** | **p_HET** |
| --- | --- | --- | --- | --- | --- | --- | --- |
| *PCCB* | 137531484 | rs1153877 | 137487615 | 1.04E-09 | 8.40E-157 | 2.86E-09 | 0.323 |
| *ABCB9* | 121979630 | rs1051434 | 122207153 | 1.42E-11 | 4.44E-23 | 2.55E-08 | 0.202 |
| *SNX19* | 130251489 | rs12420834 | 130286977 | 1.49E-08 | 1.25E-135 | 3.63E-08 | 0.147 |
| *GATAD2A* | 19478681 | rs6909 | 19480542 | 2.52E-08 | 5.12E-210 | 4.37E-08 | 0.368 |
| *NMB* | 83001462 | rs4603535 | 82974876 | 2.51E-09 | 9.60E-45 | 4.62E-08 | 0.702 |
| *PSMA4* | 76625119 | rs7164594 | 76590112 | 1.26E-08 | 1.96E-23 | 7.29E-07 | 0.449 |
| *NMRAL1* | 4451802 | rs6500601 | 4437200 | 3.73E-07 | 7.02E-83 | 8.59E-07 | 0.0572 |
| *ZDHHC5* | 57225103 | rs499188 | 57190698 | 2.75E-07 | 1.82E-48 | 1.33E-06 | 0.938 |
| *C17ORF39* | 17911918 | rs2955354 | 17889441 | 4.99E-08 | 3.63E-23 | 1.92E-06 | 0.845 |
| *RERE* | 8335369 | rs301806 | 8404665 | 1.38E-06 | 5.92E-165 | 2.10E-06 | 0.0504 |
| *SF3B1* | 197965990 | rs4685 | 197966040 | 8.20E-11 | 3.18E-11 | 3.31E-06 | 0.0639 |
| *DUS2L* | 66670652 | rs6499163 | 66800987 | 2.62E-06 | 1.85E-201 | 3.60E-06 | 0.28 |
| *C12ORF24* | 109408804 | rs7976889 | 109342077 | 1.12E-06 | 1.80E-39 | 5.21E-06 | 0.486 |
| *KCTD13* | 29825359 | rs12716973 | 29845153 | 3.68E-09 | 1.93E-12 | 6.31E-06 | 0.166 |
| *IRF3* | 54854835 | rs7251 | 54854721 | 4.06E-06 | 1.87E-109 | 7.00E-06 | 0.0635 |
| *BAG5* | 103093128 | rs2296482 | 103099572 | 3.19E-12 | 5.18E-09 | 7.69E-06 | 0.271 |
| *ENDOG* | 130622920 | rs3115874 | 130619159 | 5.34E-06 | 5.58E-107 | 8.16E-06 | 0.872 |

**Table S10. Causal genes identified by Fromer et al (*Sherlock*)**

| **Chr** | **Analyzed PGC SCZ2 GWAS regions** | **Candidate Genes** |
| --- | --- | --- |
| 1 | chr1:97792625-98559084 | *ENSG00000259946* |
| 2 | chr2:198148577-198835577  chr2:200161422-200309252  chr2:200715237-200848037 | *SF3B1*  *ENSG00000226124*  *TYW5*  *C2orf47* |
| 3 | chr3:2532786-2561686 | *CNTN4* |
| 3 | chr3:52541105-52903405 | *GLT8D1*  *NEK4* |
| 4 | chr4:170357552-170646052 | *CLCN3* |
| 6 | chr6:28303247-28712247 | *HCG17*  *VARS2*  *C4A*  *HLA-DMB*  *BRD2* |
| 6 | chr6:84279922-84407274 | *SNAP91* |
| 8 | chr8:89340626-89753626 | *ENSG00000253553* |
| 8 | chr8:143309503-143330533 | *TSNARE1* |
| 12 | chr12:123448113-123909113 | *OGFOD2* |
| 14 | chr14:103996234-104184834 | *PPP1R13B* |
| 15 | chr15:84661161-85153461 | *EFTUD1P1*  *ENSG00000259295*  *ENSG00000259726*  *ENSG00000225151* |
| 15 | chr15:91416560-91429040 | *FURIN* |
| 16 | chr16:29924377-30144877 | *INO80E*  *MAPK3* |
| 17 | chr17:17722402-18030202 | *TOM1L2*  *DRG2* |
| 19 | chr19:19374022-19658022 | *MAU2*  *GATAD2A* |
| 22 | chr22:39975317-40016817  chr22:41408556-41675156  chr22:42315744-42689414 | *NAGA*  *FAM109B*  *WBP2NL* |

**Table S11. Causal genes identified by *DEPICT***

| **Gene Name** | **Chr** | **P value** | **FDR** |
| --- | --- | --- | --- |
| *PITPNM2* | 12 | 4.13E-08 | <0.003 |
| *DGKI* | 7 | 1.03E-07 | <0.003 |
| *GRIN2A* | 16 | 5.09E-07 | <0.003 |
| *DGKZ* | 11 | 4.95E-06 | 0.013 |
| *ZNF804A* | 2 | 5.31E-06 | 0.01 |
| *NRGN* | 11 | 5.57E-06 | 0.008 |
| *NCAN* | 19 | 6.88E-06 | 0.007 |
| *KIAA1324L* | 7 | 1.12E-05 | 0.006 |
| *GRM3* | 7 | 1.16E-05 | 0.006 |
| *LRRN3* | 7 | 1.18E-05 | 0.005 |
| *HCN1* | 5 | 1.76E-05 | 0.005 |
| *KCNV1* | 8 | 1.81E-05 | 0.004 |
| *CHRM4* | 11 | 2.02E-05 | 0.004 |
| *GRIA1* | 5 | 2.15E-05 | 0.004 |
| *AKT3* | 1 | 2.34E-05 | 0.003 |
| *CSMD1* | 8 | 2.97E-05 | 0.003 |
| *C11orf87* | 11 | 4.20E-05 | 0.003 |
| *SEP3* | 22 | 5.89E-05 | 0.003 |
| *ZC3H7B* | 22 | 6.99E-05 | 0.003 |
| *BCL11B* | 14 | 7.75E-05 | 0.005 |
| *KCTD13* | 16 | 8.47E-05 | 0.005 |
| *PPP1R16B* | 20 | 8.89E-05 | 0.007 |
| *PCDHA5* | 5 | 1.29E-04 | 0.009 |
| *KCNB1* | 20 | 1.52E-04 | 0.008 |
| *PLCH2* | 1 | 1.52E-04 | 0.008 |
| *CNTN4* | 3 | 1.55E-04 | 0.008 |
| *FAM5B* | 1 | 1.55E-04 | 0.007 |
| *INA* | 10 | 2.14E-04 | 0.007 |
| *CACNA1I* | 22 | 3.55E-04 | 0.01 |
| *CALHM2* | 10 | 4.14E-04 | 0.011 |
| *PLCL1* | 2 | 4.34E-04 | 0.011 |
| *MAPK3* | 16 | 4.52E-04 | 0.011 |
| *LUZP2* | 11 | 5.26E-04 | 0.01 |
| *PCDHAC2* | 5 | 5.30E-04 | 0.01 |
| *DOC2A* | 16 | 6.64E-04 | 0.014 |
| *REEP2* | 5 | 7.21E-04 | 0.014 |
| *NGEF* | 2 | 0.001 | 0.019 |
| *RGS6* | 14 | 0.001 | 0.024 |
| *ANKRD44* | 2 | 0.002 | 0.032 |
| *MBD5* | 2 | 0.002 | 0.026 |
| *GRAMD1B* | 11 | 0.002 | 0.032 |
| *RIMS1* | 6 | 0.002 | 0.024 |
| *IGSF9B* | 11 | 0.002 | 0.034 |
| *PLCB2* | 15 | 0.002 | 0.033 |
| *DRD2* | 11 | 0.002 | 0.031 |
| *FUT9* | 6 | 0.002 | 0.032 |
| *PPP1R13B* | 14 | 0.003 | 0.04 |
| *R3HDM2* | 12 | 0.003 | 0.04 |
| *PCDHA10* | 5 | 0.003 | 0.041 |
| *SNAP91* | 6 | 0.003 | 0.04 |
| *MMP16* | 8 | 0.004 | 0.042 |
| *SLC45A1* | 1 | 0.004 | 0.042 |
| *TMEM22* | 3 | 0.004 | 0.041 |
| *ASPHD1* | 16 | 0.004 | 0.04 |
| *GPM6A* | 4 | 0.005 | 0.047 |
| *TRANK1* | 3 | 0.005 | 0.047 |
| *TCF4* | 18 | 0.005 | 0.046 |


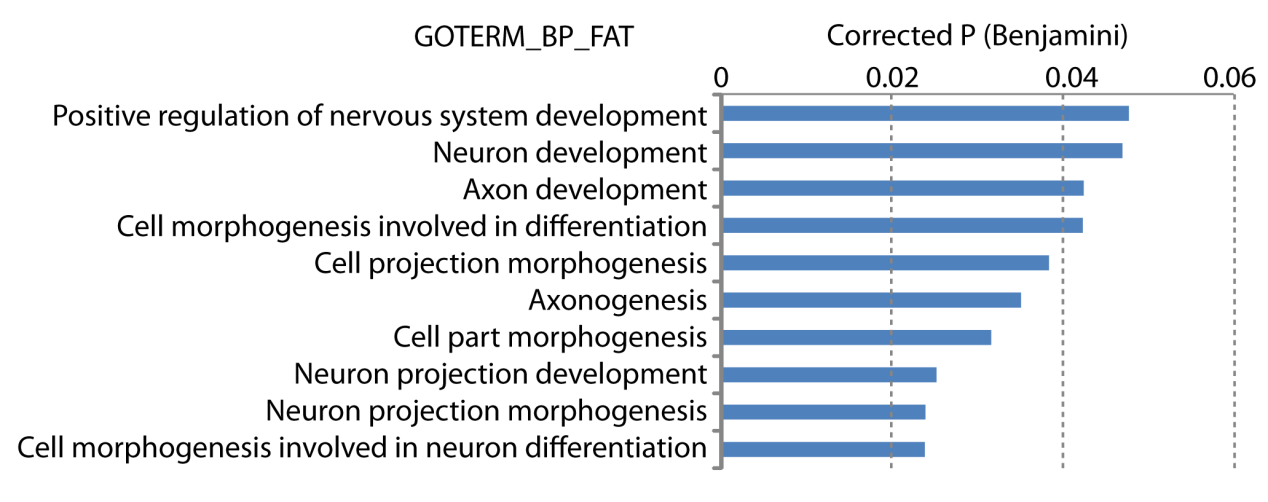


**Figure S1. Gene Ontology (GO) analysis showed that neural development and neural projection categories were enriched in the causal genes prioritized by *NetWAS*.**

**
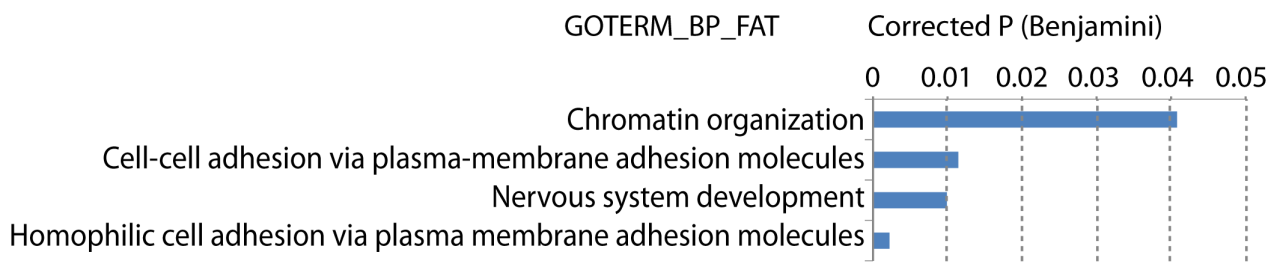
**

**Figure S2. Gene Ontology (GO) analysis showed that nervous system development and cell-cell adhesion categories were enriched in the causal genes prioritized by *DAPPLE*.**

**
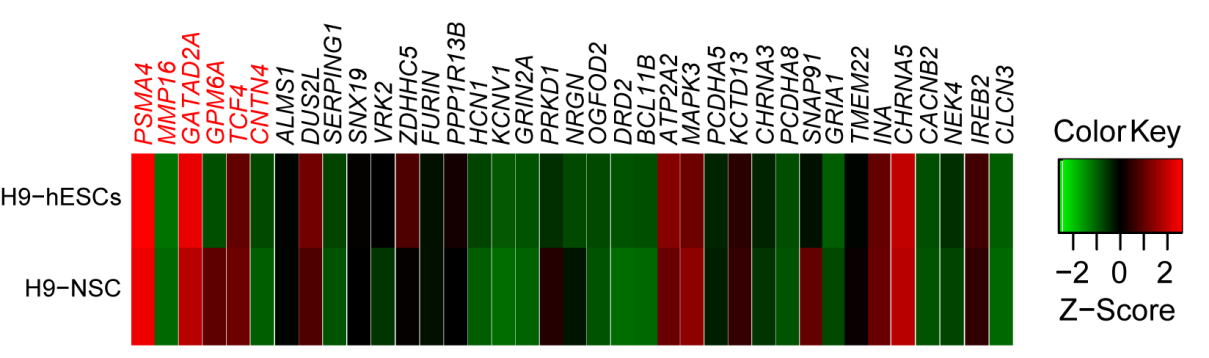
**

**Figure S3. Top causal genes were highly expressed in human embryonic stem cells (hESCs) and neural stem cells (NSCs).**

**
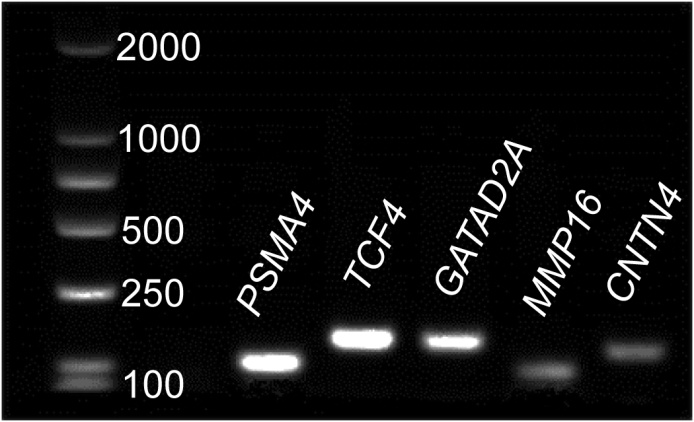
**

**Figure S4. RT-PCR showed that top causal genes (*PSMA4, TCF4, GATAD2A, MMP16 and CNTN4*) were expressed in SH-SY5Y cells.**

**
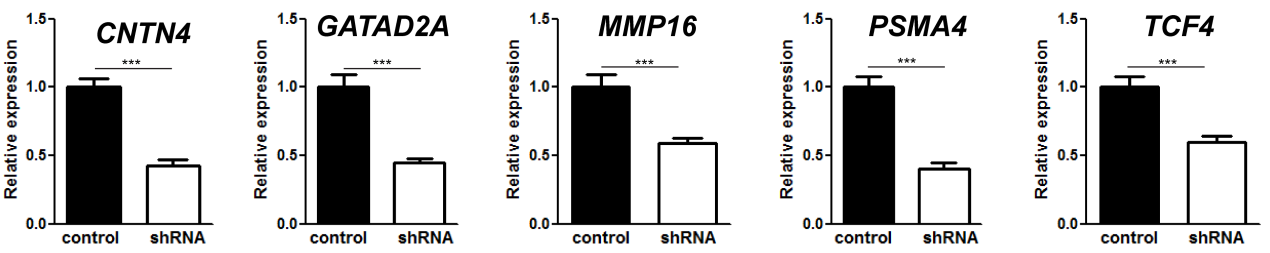
**

**Figure S5.** Short hairpin RNAs significantly down-regulated the expression of *CNTN4, GATAD2A, MMP16, PSMA4* and *TCF4* genes in SH-SY5Y cells. ****P*<0.001.

**
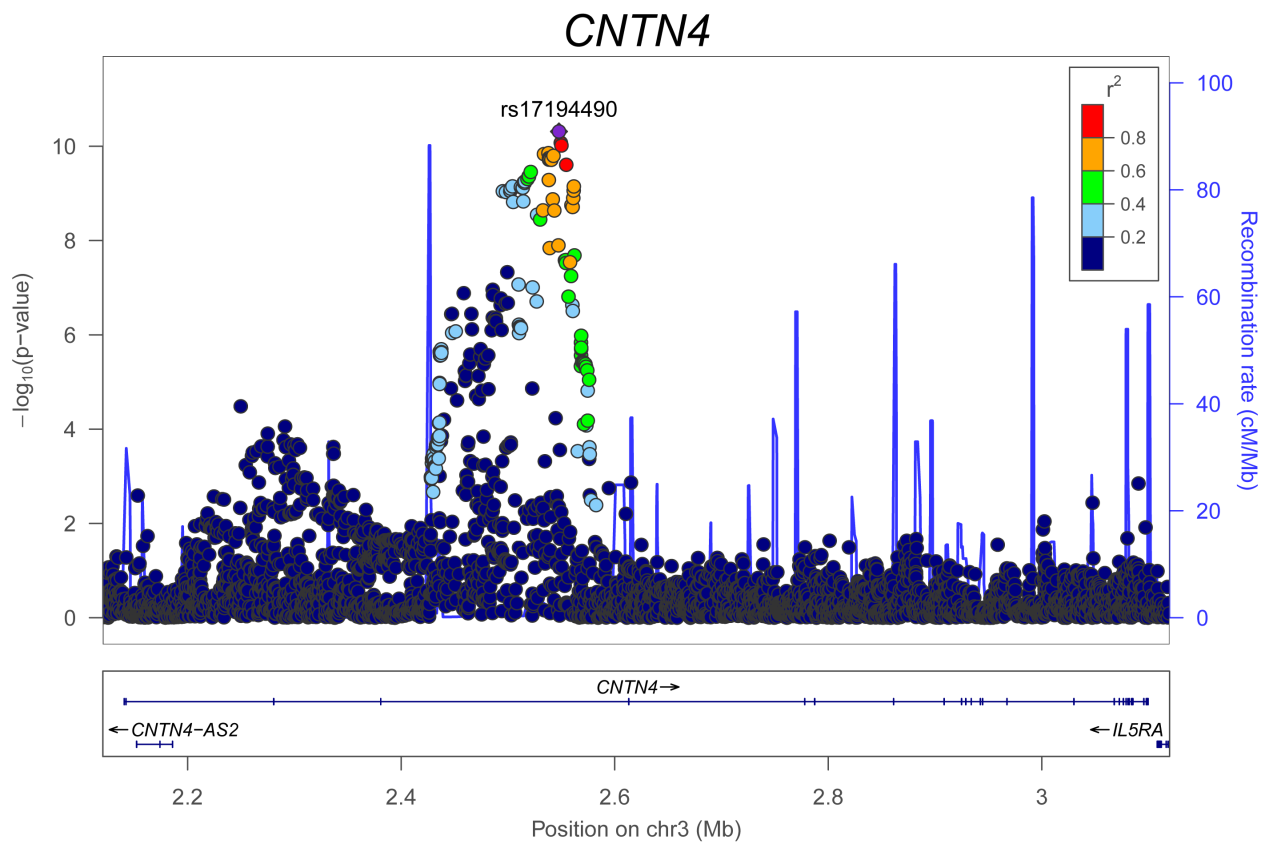
**

**Figure S6. *CNTN4* showed genome-wide significant association with schizophrenia (data from PGC2).**

**
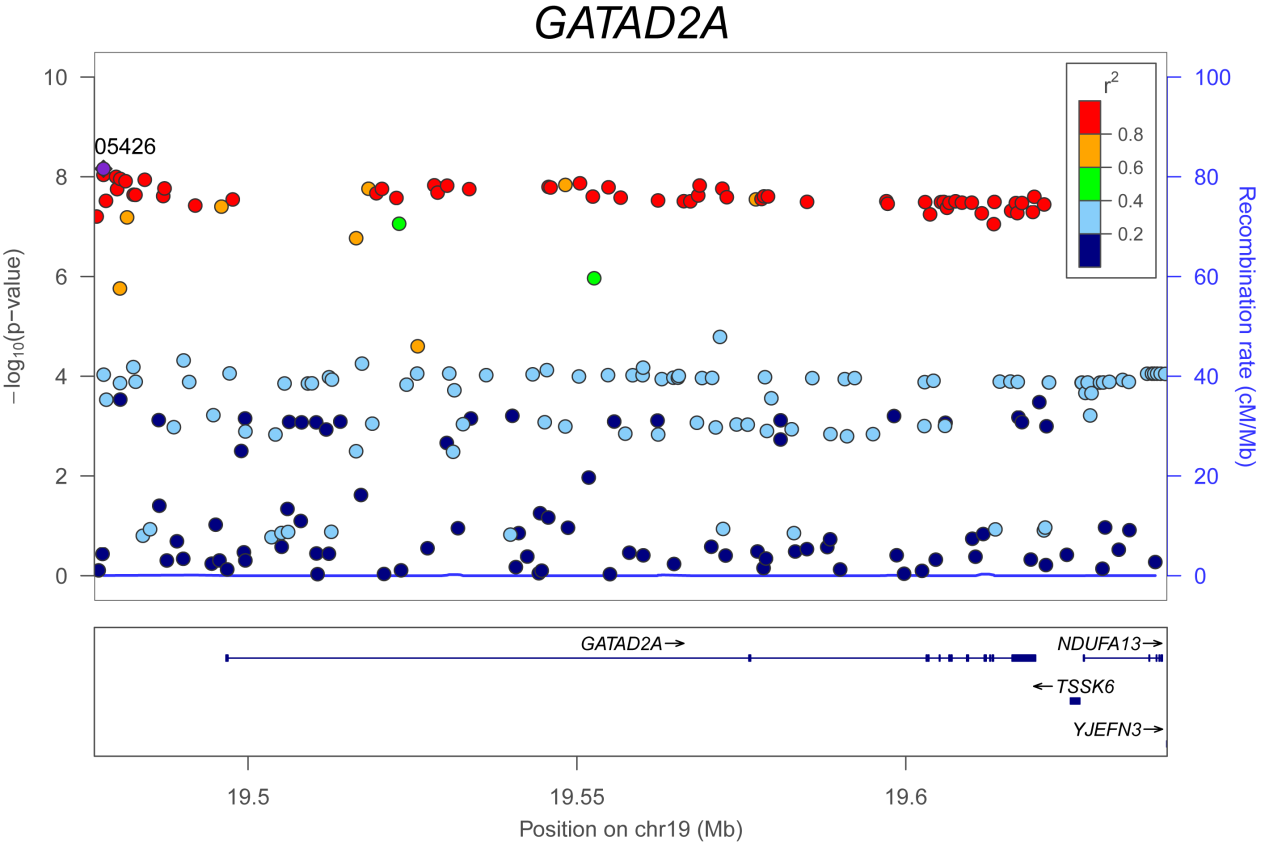
**

**Figure S7. *GATAD2A* showed genome-wide significant association with schizophrenia (data from PGC2).**

**
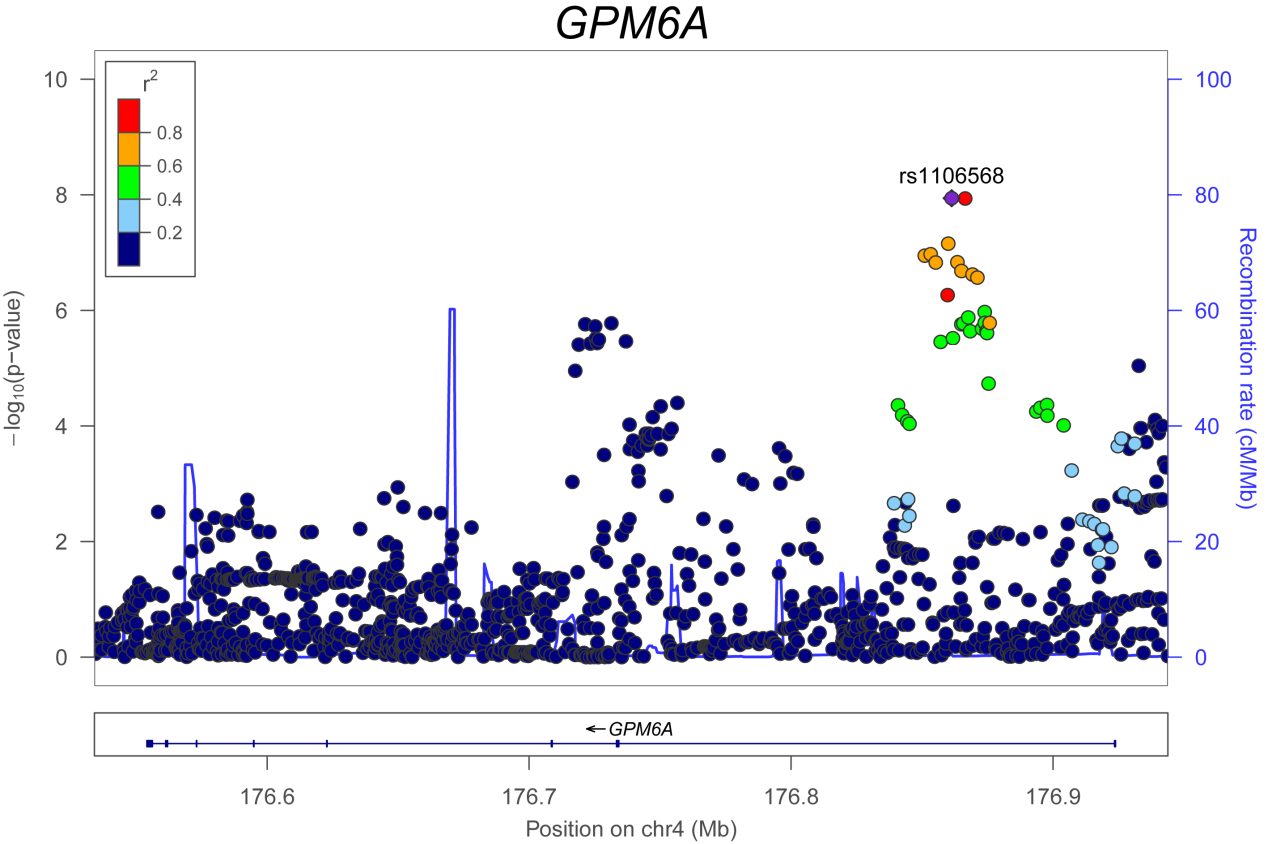
**

**Figure S8. *GPM6A* showed genome-wide significant association with schizophrenia (data from PGC2).**

**
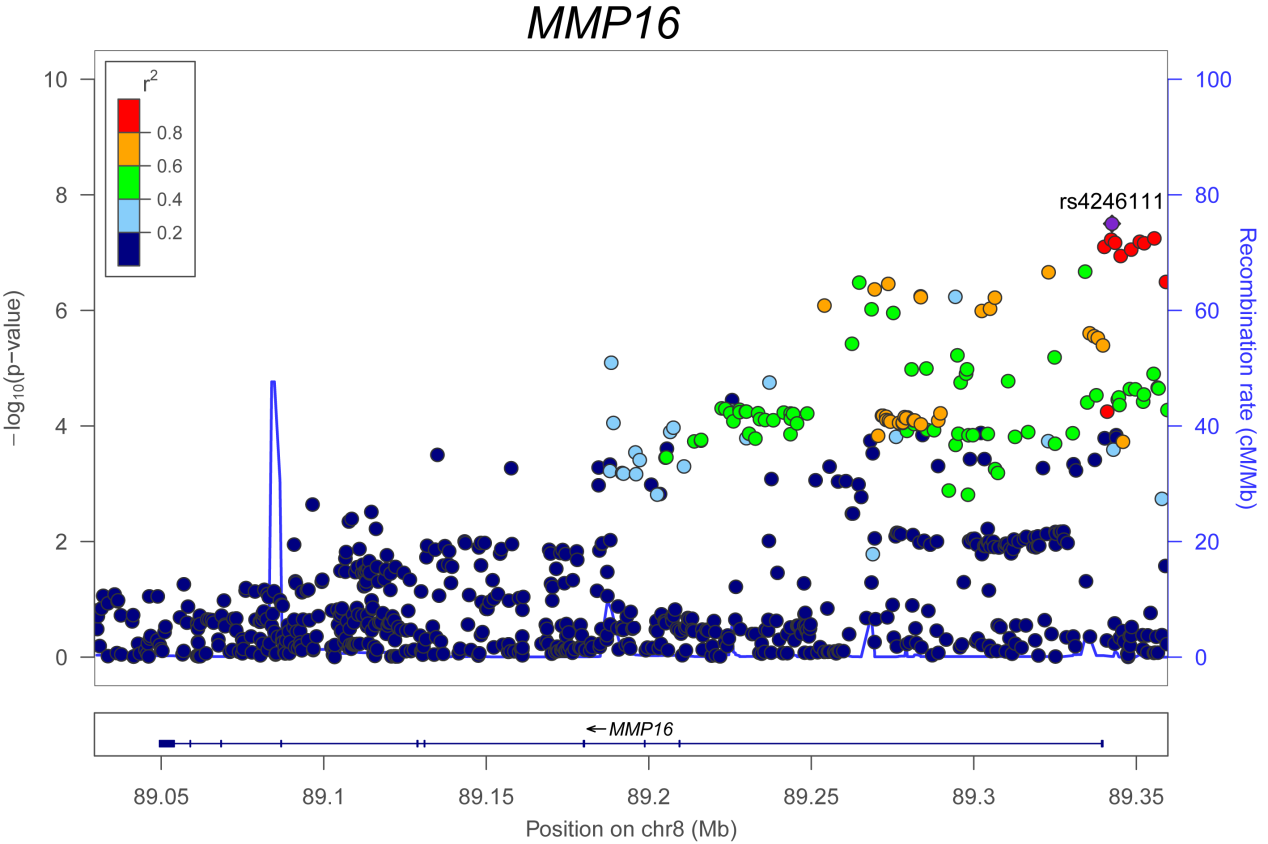
**

**Figure S9. *MMP16* showed genome-wide significant association with schizophrenia (data from PGC2).**

**
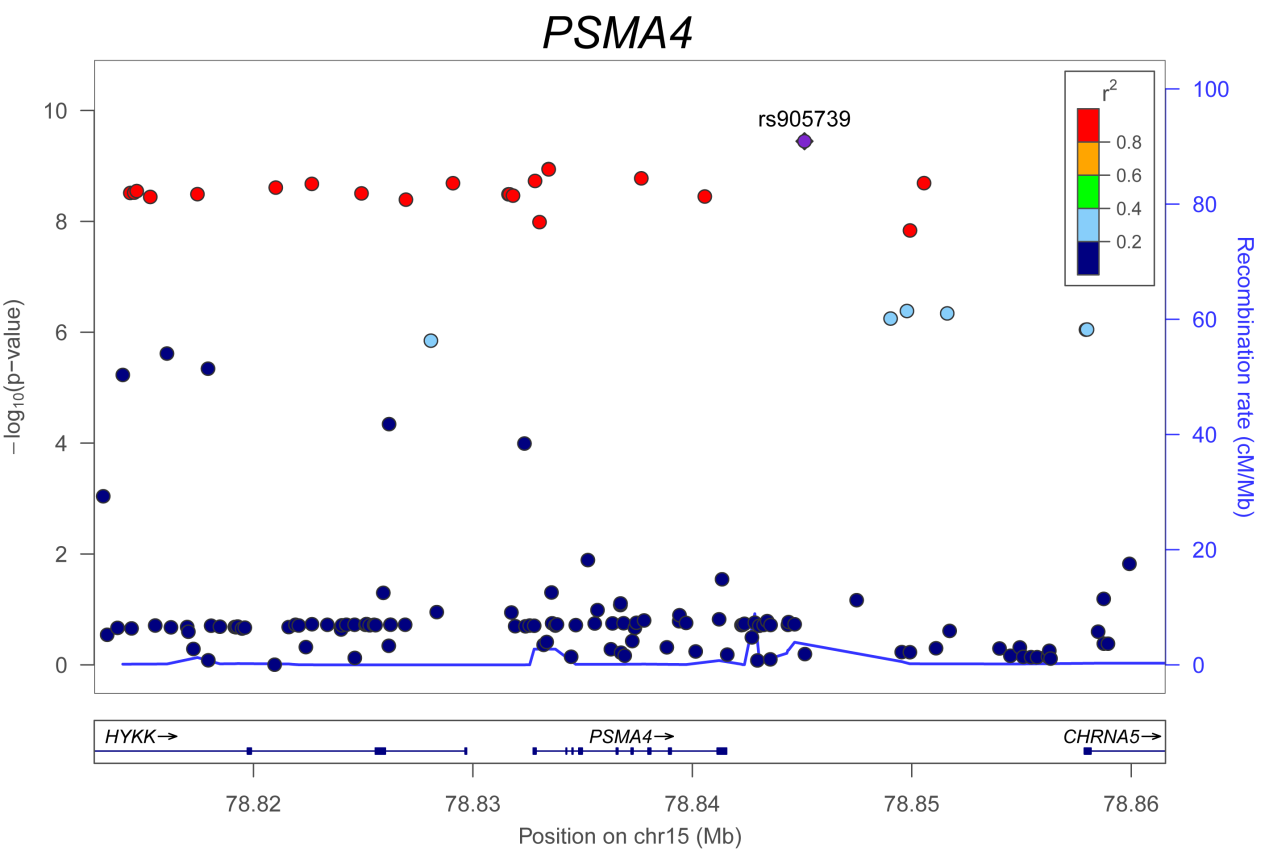
**

**Figure S10. *PSMA4* showed genome-wide significant association with schizophrenia (data from PGC2).**

**
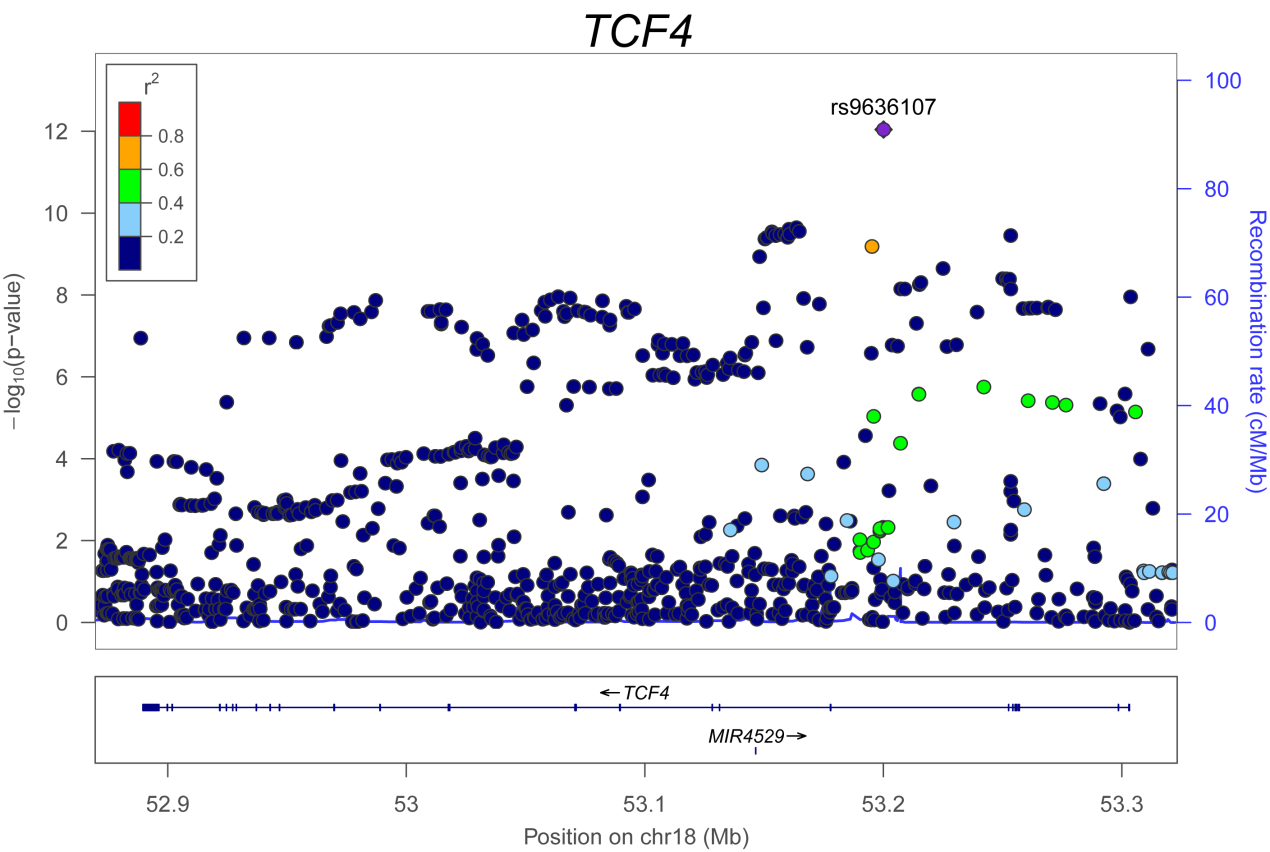
**

**Figure S11. *TCF4* showed genome-wide significant association with schizophrenia (data from PGC2).**

**References**

1 Ong CT, Corces VG. Enhancer function: new insights into the regulation of tissue-specific gene expression. *Nat Rev Genet* 2011; **12**: 283-293.

2 Schizophrenia Working Group of the Psychiatric Genomics Consortium*. Biological insights from 108 schizophrenia-associated genetic loci. *Nature* 2014; **511**: 421-427.

3 Fatemi SH, Folsom TD. The neurodevelopmental hypothesis of schizophrenia, revisited. *Schizophr Bull* 2009; **35**: 528-548.

4 Owen MJ, O'Donovan MC, Thapar A, Craddock N. Neurodevelopmental hypothesis of schizophrenia. *Br J Psychiatry* 2011; **198**: 173-175.

5 Myers AJ, Gibbs JR, Webster JA, Rohrer K, Zhao A, Marlowe L *et al.* A survey of genetic human cortical gene expression. *Nat Genet* 2007; **39**: 1494-1499.

6 Zhu Z, Zhang F, Hu H, Bakshi A, Robinson MR, Powell JE *et al.* Integration of summary data from GWAS and eQTL studies predicts complex trait gene targets. *Nat Genet* 2016; **48**: 481-487.

7 Westra HJ, Peters MJ, Esko T, Yaghootkar H, Schurmann C, Kettunen J *et al.* Systematic identification of trans eQTLs as putative drivers of known disease associations. *Nat Genet* 2013; **45**: 1238-1243.
